# 3D morphology of the Cambrian bivalved arthropod *Sunella* informs about head segmentation, arthrodization, and arthropodization

**DOI:** 10.1038/s42003-026-09909-z

**Published:** 2026-03-21

**Authors:** Cong Liu, Stephen Pates, Mingjing Zhang, Yu Wu, Jiaxin Ma, Dongjing Fu, Xingliang Zhang

**Affiliations:** 1 State Key Laboratory of Continental evolution and Early Life, Shaanxi Key Laboratory of Early Life and Environments, Department of Geology, Northwest University, Xi’an, China; 2 Centre for Ecology and Conservation, University of Exeter—Penryn Campus, Penryn, Cornwall, UK; 3 Department of Earth Sciences, University College London, London, UK; 4 Centre for Life’s Origins and Evolution, Department of Genetics, Evolution and Environment, University College London, London, UK; 5 Nanjing Institute of Geology and Paleontology, Chinese Academy of Sciences, Nanjing, China

**Keywords:** Palaeontology, Taxonomy

## Abstract

A head region with specialised appendages, sclerotization, and segmentation of the trunk (arthrodization), and its appendages (arthropodization) represent three key innovations in arthropod evolution. Two scenarios have been proposed for acquisition of these innovations either in a synchronous or a sequential mode. Here we describe a new species *Sunella dimorphismus* sp. nov., from the Chengjiang biota (ca. 518 Ma), which displays a bivalved carapace, raptorial frontal appendages, and an arthrodized trunk with a series of biramous arthropodized appendages revealed by new specimens and new observations with assistance of computed tomography. Phylogenetic analyses placed *Sunella* as the earliest diverging deuteropods besides *Erratus*. Ancestral state reconstructions refine our understanding of the gain and loss of key characters in the euarthropod. The results demonstrate that trunk limb arthropodization preceded trunk arthrodization, with both prior to the evolution of a six-segmented functional head, while the trunk arthrodization is found to be lost in isoxyiids.

## Abstract (web summary)

Three-dimensional preservation of the Cambrian arthropod *Sunella* reveals new details of head segmentation and limb organisation, informing early stages of arthropod body-plan evolution.

## Introduction

The evolution of sclerotized and articulated appendages (arthropodization), a segmented and sclerotized body (arthrodization), and a discrete head region separated from a trunk with specialised appendages (cephalization) represent three critical characteristics of the Euarthropoda—a widely diverse group that includes chelicerates, myriapods, and pancrustaceans (including insects)^1–3^.

However, despite their importance for the ecological success and diversification of euarthropods since the Cambrian, the exact origins and sequence of acquisition of these features remains contested^3–7^, and depend on the taxa or groups determined as the earliest diverging deuteropods (euarthropods with a multisegmented head carrying structurally differentiated deutocerebral appendages and reduced protocerebral appendages^3^). Radiodonts—a group of Cambrian apex predators including *Anomalocaris*—are confidently identified as the earliest diverging members of the euarthropod. Radiodonts are also the first euarthropods possessing arthropodized appendages, represented by a single pair of protocerebral feeding appendages attached to the head^4,8^. However, three candidates, *Erratus, Kylinxia* and *Parapeytoia*, are currently considered as the possible earliest diverging candidates of the Deuteropoda (Table 1), in the node immediately crownwards of radiodonts. Each candidate provides different implications for the origins and relative timings of euarthropod innovations. The *Kylinxia* hypothesis (with or without *Fengzhengia mamingae*) suggests an origin of the arthropodized trunk limb, arthrodized trunk, and a six-segmented head at the base of Deuteropoda^9,10^ and implies a synchronous acquisition. Alternatively, euarthropods with bivalved carapaces covering their body, *Erratus, Isoxys*, and/or *Surusicaris* have been placed in this position^11–13^. In this scenario, the arthropodized trunk limb is thought to have originated earlier than the arthrodized body, and the head of the ancestral deuteropod had six segments (indicated by four small pairs of appendages behind the deutocerebral raptorial appendage pair in *Isoxys*^10,13^). *Parapeytoia yunnanensis* has also been proposed as the sister taxon to all other members of the euarthropods (except radiodonts)^6^, but not as a deuteropod *sensu* ref. ^3^. In this interpretation, most of the early diverging euarthropod groups (including *Parapeytoia* and megacheirans) only had a protocerebral brain and a biramous limb with gnathobases evolved prior to dorsal arthrodization^6^. Numerous phylogenetic analyses using a range of matrices have not yet reached a consensus^6,9–17^, while none of these analyses includes all of *Erratus, Isoxys, Kylinxia*, and *Parapeytoia* as terminals in their analyses (Table 1^6,9–20^).

**Table 1:** Recent phylogenetic analyses (since 2020) considering the earliest deuteropod

Study	Earliest diverging deuteropod(s) recovered	Includes *Erratus*?	Includes isoxyiids (*Isoxys +/- Surusicaris*)	Includes *Kylinxia*?	Includes *Parapeytoia*?	Matrix derived from
Zeng et al. *Nature*^9^	*Kylinxia zhangi*	-	Y	Y	N	Zeng et al. *Nature*^9^
O’Flynn et al. *Paleontologia Electronica*^14^	*Kylinxia zhangi*	-	Y	Y	N	Zeng et al. *Nature*^9^
O’Flynn et al. *Current Biology*^10^	*Kylinxia zhangi + Fengzhengia mamingae*	N	Y	Y	N	Zeng et al. *Nature*^9^
O’Flynn et al. *Papers in Palaeontology*^15^	*Kylinxia zhangi + Fengzhengia mamingae*	N	Y	Y	N	Zeng et al. *Nature*^9^
Fu et al. *Trans R Soc B*^12^	*Erratus sperare*	Y	Y	N	N	Fu et al. *Trans R Soc B*^12^
Pates et al. *Nature Communications*^a^^16^	Parapeytoia yunnanensis*	-	N	Y	Y*	Pates et al. *Proc R Soc B*^18^
Zhang et al. *Proc R Soc B*^13^	*Erratus sperare* (MP) or *Surusicaris elegans* (BI)	Y	Y	Y	N	Pates et al. *Proc R Soc B*^18^
Aria *Paleobiology*^11^	*Isoxys* spp. + *Surusicaris elegans*	-	Y	-	Y	Aria *Paleobiology*^11^
Izquierdo Lopez & Caron *Royal Society Open Science*^b^^17^	*Erratus sperare*	Y	Y	Y	N	Aria *Paleobiology*^11^
Jin et al. *Papers in Palaeontology*^c^^19^	*Surusicaris elegans*	-	Y	N	N	Yang et al. *Nature Communications*^20^

^a^This study was not strictly interrogating the earliest deuteropod, but a number of sensitivity analyses were conducted to address whether the inclusion of different deuteropods (*Kylinxia, Parapeytoia*) impacted the results. Results presented in this table are from the sensitivity analyses considering *Parapeytoia* appendages and deutocerebral, see Supplementary Fig. 11 in Pates et al. *Nature Communications*.

^b^This study was not strictly interrogating the earliest deuteropod. It is included here as it used a different base matrix, and includes *Erratus, Isoxys*, and *Kylinxia* as terminals in the analysis.

^c^This study was not interrogating the earliest deuteropod. It is included here as it used a different base matrix, and includes *Isoxys* as terminals in the analysis.

Earliest deuteropod recovered // Study // Includes *Erratus*? // Includes *Parapeytoia*? // Includes *Kylinxia*? // Includes *Isoxys*? // Matrix derived from

Y = included; N = not included, - = not included because taxon not yet published or was published in same year; *BI* Bayesian Inference, *MP* Maximum Parsimony.

Here we describe a new species of the bivalved euarthropod *Sunella dimorphismus* sp. nov., which has previously been considered a bradoriid^21^, a relative of isoxyiids^22^, or in a more open position within euarthropods^23^. New material and new observations, combined with computed tomography (CT) imaging technique, reveal additional details of its frontal appendages, eyes, tagmatization, and the segmented appendages of the thorax. Phylogenetic analyses, including *Erratus*, isoxyiids, *Kylinxia* and *Parapeytoia* as terminals, resolve the acquisition sequence of key euarthropod characters, suggesting that trunk limb arthropodization evolved prior to arthrodization, both of which arose prior to a six-segmented functional head within the euarthropod crown lineages.

## Results

### Systematic palaeontology

Phylum Euarthropoda Lankester^24^

Class and Order Uncertain

Family Sunellidae Huo^25^

#### Genera included

*Sunella* Huo^25^; *Caudicaella* Sun et al.^26^; *Jinningella* Huo and Shu^27^ and *Combinivalvula* Hou^28^.

Genus *Sunella* Huo^25^

*Type species*: *Sunella grandis* Huo^25^.

#### Discussion

Zhang and Shu^23^ emended the diagnosis of the *Sunella* characterised by the presence of a dimorphic carapace, a hinge line across the whole carapace, an anterodorsal sulcus projecting from the anterodorsal angle to the anteromedian part of the valve, and the lack of ornamentation. *Sunella* is here considered to include two species from South China, *Sunella grandis* Huo, and *Sunella dimorphismus* sp. nov. (details in Supplementary information).

### *Sunella dimorphismus* sp. nov

**Fig. 1:**
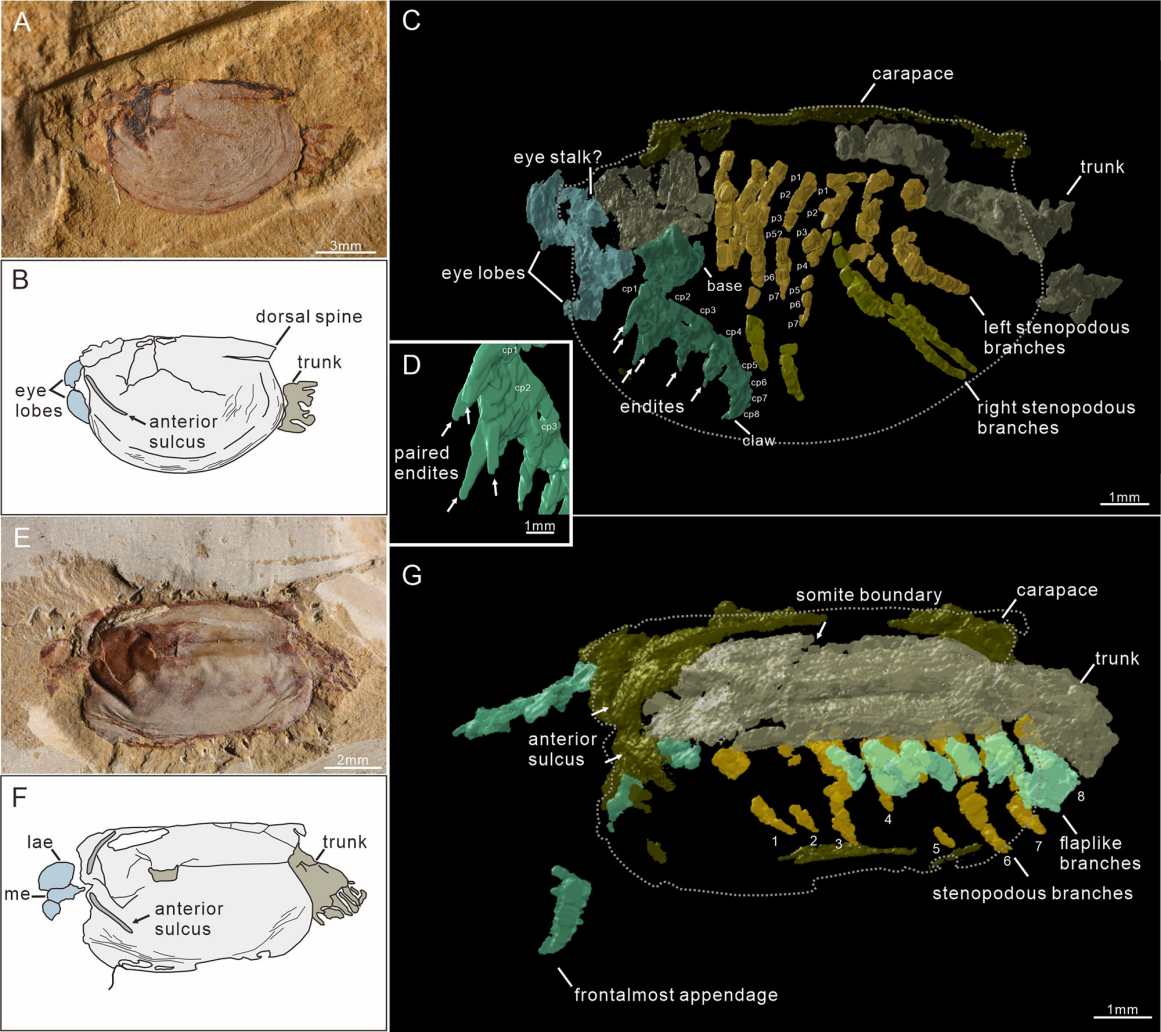
Micro-CT scanning of *Sunella dimorphismus.* **A**–**D** SJZ-B10-753A in lateral view. **A** general view. **B** Camera-lucida drawing of *A*. **C** Rendering model; showing a pair of stalked eyes, a radiodont-like frontal appendage composed of a shaft with at least eight podomeres and a terminal claw, and paired trunk stenopodous branches with seven podomeres. **D** Close-up of claw endites, showing paired En1 and En2. E-G, JS-274A in dorsal view. **E** General view, showing a pair of lateral eyes, a median eye, and a partial trunk projecting from the posterior margin of the carapace. **F** Camera-lucida drawing of *E*. **G** rendering model, showing a pair of frontal appendages projecting from the anterior margin of the carapace, homogeneous trunk appendages with segmented and flaplike branches. cp1-8: 1st–8th claw podomere; p1-7: 1st–7th podomere of stenopodous branch; lae: lateral eyes; me: median eye.

**Fig. 2:**
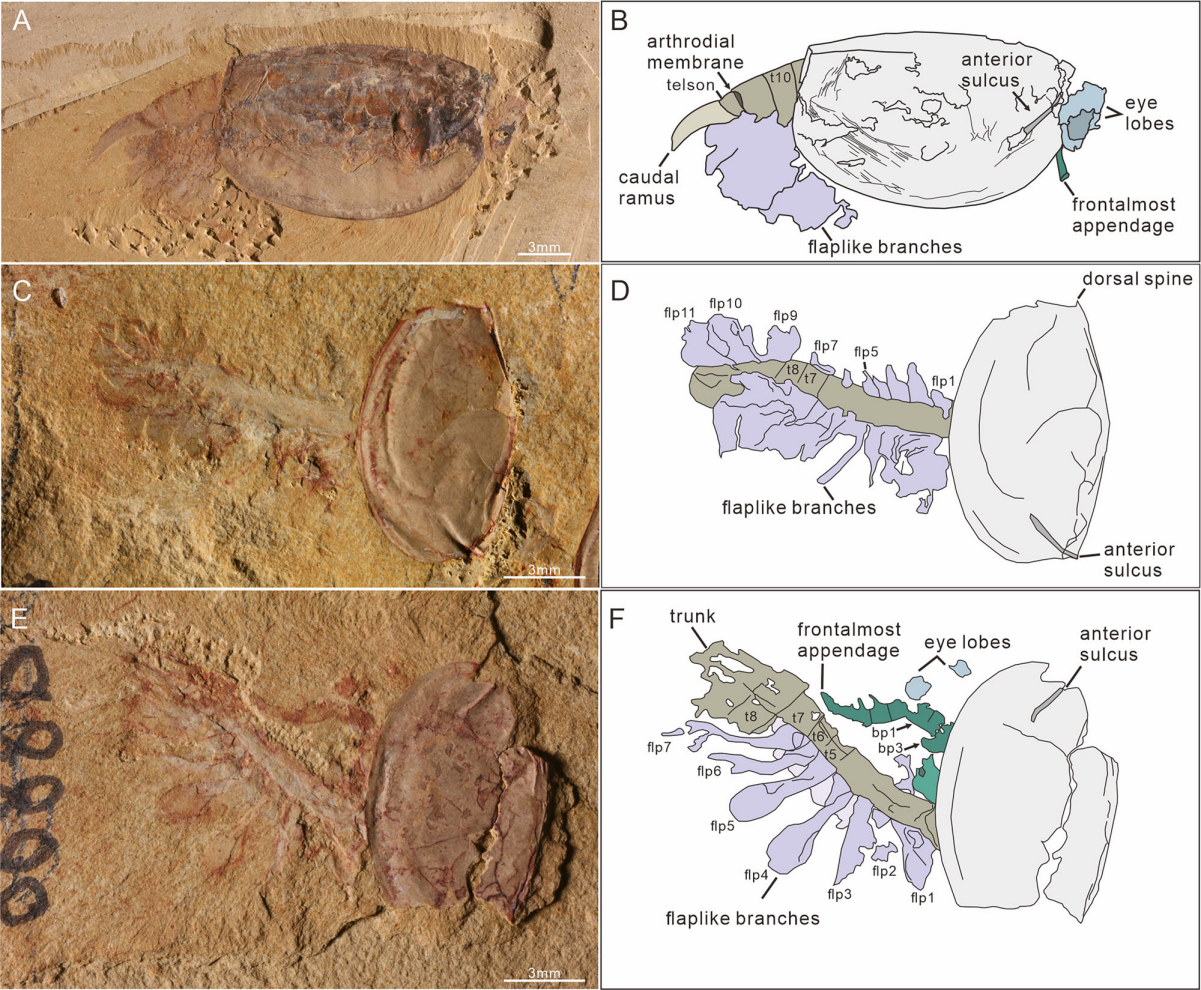
Trunk segmentation and appendicular structures. **A** TLP-064, specimen in dorsal view, showing carapace with a subelliptical outline, three somites (9th–11th) projecting from the posterior margin of the carapace, a short ring-shaped telson, a flexible arthrodial membrane between the 11th somite and telson, and a paddle-shaped caudal ramus. **C** SUN 0090, specimen with carapace rotated nearly 90° anterodorsally to the internal body axis, showing the 7th and 8th segmental boundaries as straight lines and 11 flap-like appendages arranged along the left side of the body. **E** SUN 0097 A, a specimen with a rotated carapace, showing a radiodont-like frontal appendage with a 3-podomerous shaft, the boundaries of the 5th–8th somites, a flapped branch with a sub-ovoid outline, and ramified tissue traces within the flap appendages. **B**, **D**, **F** Camera-lucida drawings of **A**, **C**, **E**, respectively. bp1-3: 1st–3rd basal (shaft) podomere; flp1-11: 1st–11th flaplike appendages; t5-8: 5th–8th trunk somite.

**Fig. 3:**
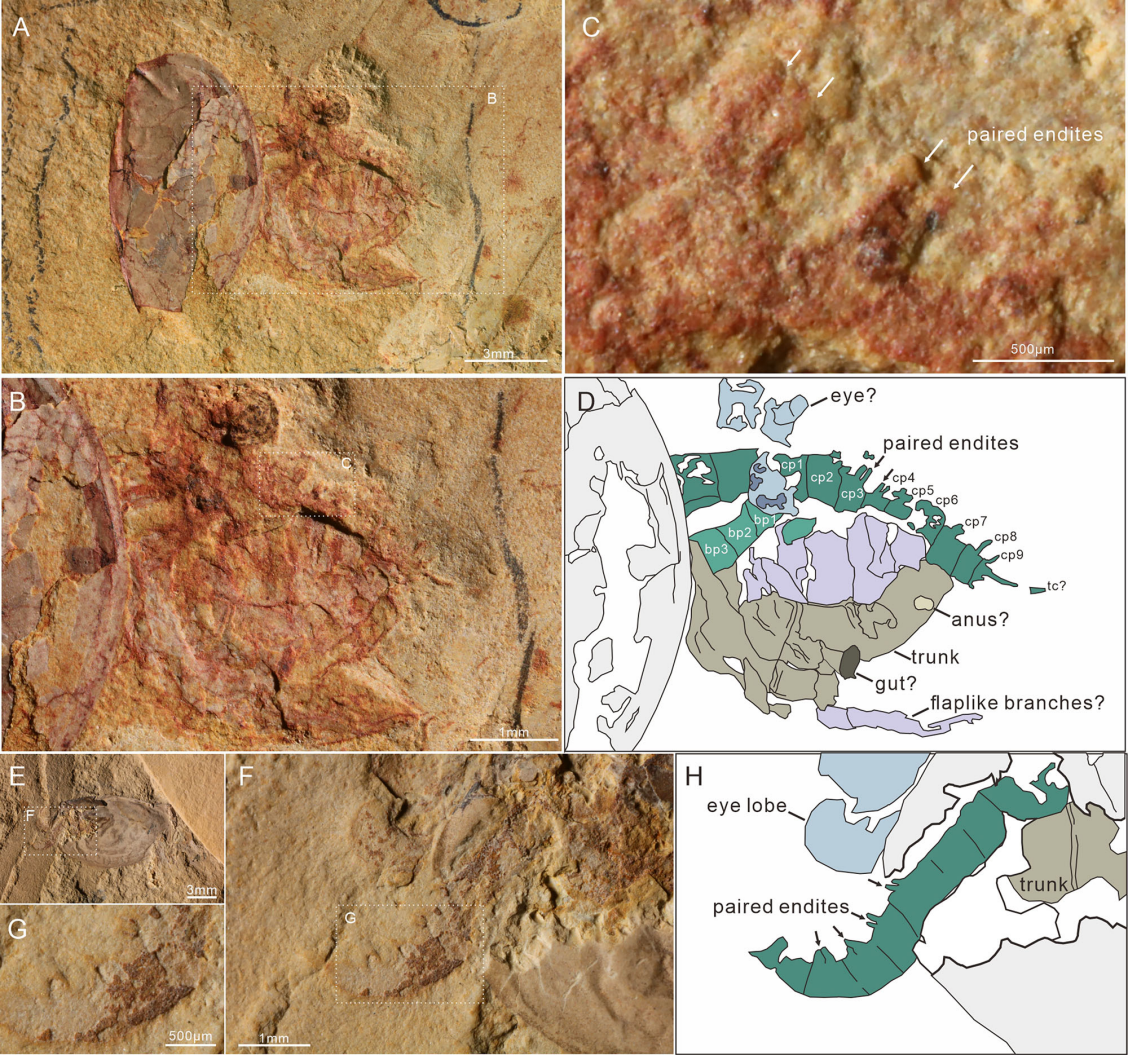
Details of the radiodont-like frontal appendage. **A**–**C** SUN 0073 A, specimen in lateral view with a 90° rotated carapace. **B** Enlargement of soft tissues, showing a 3-podomerous shaft, a 9-podomerous claw with paired endites (close-up in **C**), flapped appendage traces, and trunk. **E**–**G** SUN 0043, lateral view. **F** Enlargement of radiodont-like frontal appendage, showing it is located immediately posterior and adjacent to the stalked eyes. **G** Close-up of paired claw endites. **D**, **H** Camera-lucida drawings of **B**, **F**, respectively. tc terminal claw.

**Fig. 4:**
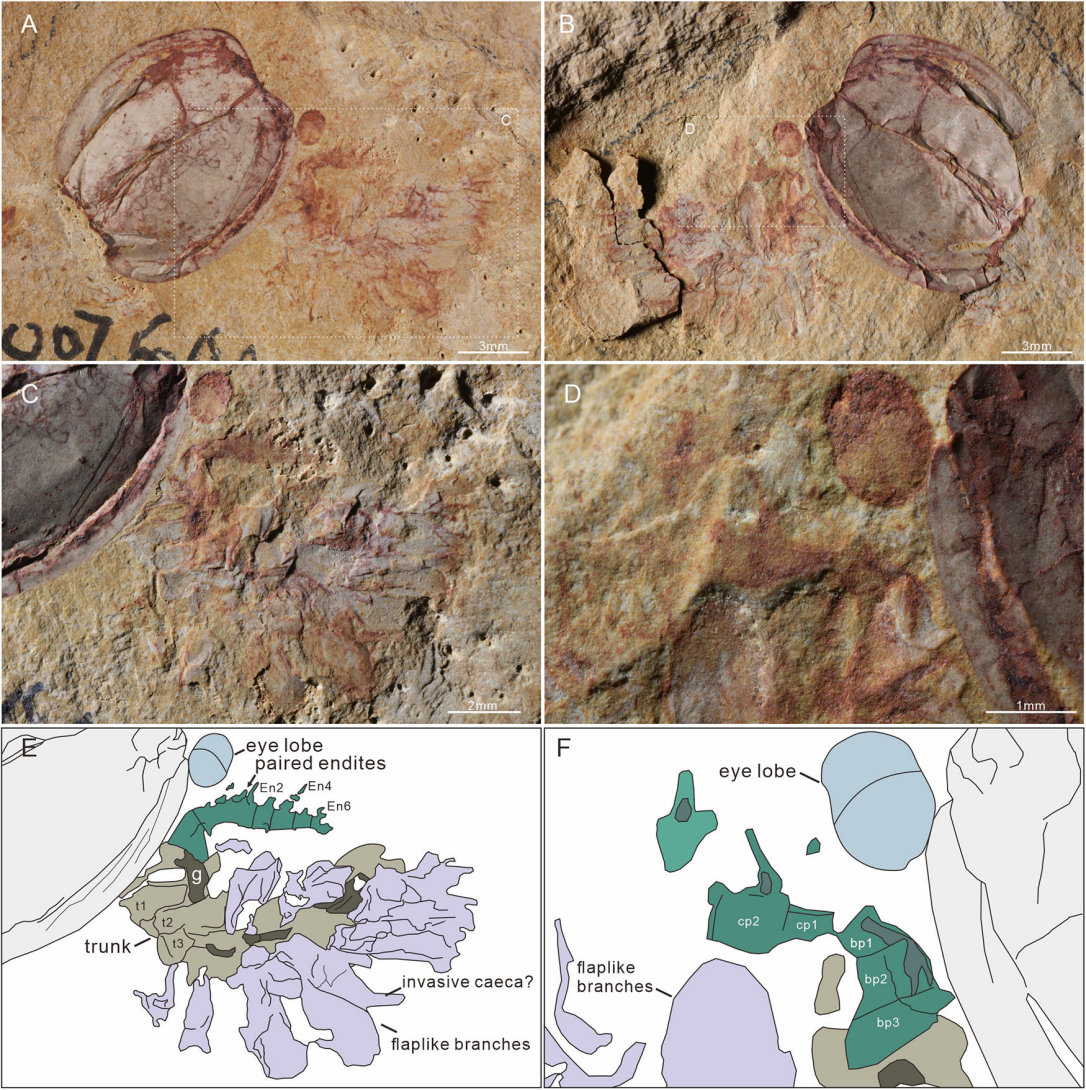
Appendicular structures in specimen SUN 0076. **A**, **B** General view of SUN 0076 A and SUN 0076B, respectively. Showing a specimen in the dorsal view with a strong rotation of the carapace. **C**, **D** Low-angle lighting images, with enlargement of the soft tissues in (**A**) and the frontal appendages in (**B**), respectively. Showing a 3-podomerous shaft, a long distal claw with endites, an obtuse angular of the dorsal surface between the base and claw, the boundaries of the 1st–3rd somites, and a flapped branch with ramified tissue traces. **E**, **F** Camera-lucida drawings of **C**, **D**, respectively. En2, 4, and 6: 2nd, 4th, and 6th claw endite.

Synonymy: 2007 *Sunella* cf. *shensiensis* (Huo, 1965), Zhang and Shu^23^, Figs. 3; 4, 1–4; 6.

#### Diagnosis

*Sunella* with an elongate, dimorphic bivalved carapace covering the head and thorax. Valves elliptical in lateral view; hinge line dorsal; short cardinal spines present anteriorly and posteriorly; anterodorsal sulcus extending to anteromedian of the valve; anterior and posterior margins diverging from the dorsal margin at ca. 120°; doublure narrow. Body of 14 segments, divided into head (two segments), thorax (eight segments), and abdomen (four segments). Head bearing a pair of large stalked eyes, a small median eye, and a pair of frontal appendages consisting of three base podomeres and nine claw podomeres (Cp); Cp1–Cp9 bearing paired spinose endites. Thorax carrying eight pairs of homogeneous appendages with a seven-segmented stenopodous branch and a flap-like branch. Abdomen bearing three pairs of flap-like appendages and a flattened caudal structure.

#### Remarks

The specimens herein assigned to *Sunella dimorphismus* sp. nov. are synonymous with the material assigned by Zhang and Shu^23^ to *Sunella cf. shensiensis* (Huo). Both exhibit similar carapace shapes (Supplementary Fig. 1) with elongate valves, narrow doublure, short cardinal spines, curved dorsal margins and enlarged dorsal angles—falling in the same area of PC space following elliptical Fourier analysis (Supplementary Fig. 2; full details section Outline Analysis in Supplementary information). *Sunella shensiensis* Huo^25^ (known only from its holotype) is synonymized with *S. grandis* (see details section Comparisons between *Sunella* species in Supplementary information), which can be distinguished from *S. dimorphismus* by carapace shape. Therefore, *Sunella shensiensis* is considered as invalid taxon and a new species, *Sunella dimorphismus* sp. nov. is herein established, including specimens previously attributed to *Sunella* cf. *shensiensis*^23^ and new specimens presented in this study.

#### Occurrence

*S. dimorphismus* occurs in the *Eoredlichia*-*Wutingaspis* assemblage zone of the Yu’anshan shale Member (Helinpu Formation), Cambrian Series 2, Stage 3. Specimens described here were collected from the five localities (Chengjiang, Dapoutou, Jianshan, Sanjiezi, and Tanglipo sections) of Eastern Yunnan, China.

#### Description

Based on 30 new and 22 prior specimens^23^ of *S. dimorphismus* analyzed via photography, microscopy, and CT, we revise its carapace to cover only the head and thorax, documenting four new features: three tagmata, paired lateral eye stalks, dorsally-curving arthropodized frontal appendages, and stenopodous thoracic branches with at least 7 segments.

#### Carapace

Thirty new specimens show features consistent with those described in the previous work^23^. Ten specimens, seven in the dorsal view (Fig. 1D; Supplementary Fig. 3C) and three in the lateral view (Figs. 1A and 2A), illustrate an abdomen extending from the posterior aspect of the carapace. It is clear from the lateral view TLP-064 that the carapace covers approximately the anterior two-thirds of the body, with four abdominal segments and a caudal ramus projecting from the posterior margin of the carapace (Fig. 2A, B).

#### Tagmata

The body can be divided into three broad regions: (1) a head encompassing stalked eyes, a single median eye, and a pair of raptorial appendages (Figs. 2E and 3); (2) a thorax of eight segments bearing paired homonomous biramous appendages; (3) an abdomen beyond the carapace, which is composed of three segments with flaplike appendages only and a telson bearing an ovoid, paddle-shaped caudal ramus. Segmental boundaries of the trunk are straight or slightly curved lines. The boundaries of the 1st–3rd trunk segments can be seen in specimen SUN 0076B, where the body is twisted and preserved in the dorsal aspect (Fig. 4A, C). The curved posterior segmental boundary of the 3rd trunk segment is also observed in the Micro-CT scanning data of JS-274A (Fig. 1G). The boundaries of the 5th–11th trunk segments are observed in four specimens (one in dorsal view and three in lateral view; Fig. 2C–F; Supplementary Fig. 3C, D). The 11th trunk segment is connected with the telson by a flexible arthrodial membrane in TLP-064 (Fig. 2A, B). The length of this ring-shaped telson is approximately half that of the 11th segment (Fig. 2A, B).

#### Eyes

Paired lateral eyes are observable in 20 specimens, 11 of which are newly reported here. In these specimens, the eye lobe extends beyond the anterior margin of the carapace. It is oval in shape, with a maximum diameter of 2.1 mm and a long axis of 2.6 mm (about 17% of the carapace length). The paired lateral eye lobes in three specimens (TLP-064, SJZ-B18-871, and SUN 0095, as depicted in Fig. 4.1 of Zhang and Shu’s article^23^) consist of a light outer layer and a dark internal mass (Fig. 2A; Supplementary Fig. 3A; 4D, F). Stalked lateral eyes are confirmed in specimen SUN 0100 (Supplementary Fig. 4A–C), which exhibits a large eye lobe on a stout, cylindrical stalk (0.6 mm diameter, 0.4 mm long). Micro-CT data from SJZ-B10-753A further reveal these stalks beneath the carapace (Fig. 1C).

A well-preserved, central median eye in three new specimens confirms its position within the ocular unit, supporting previous observation^23^ (Fig. 1E, F; Supplementary Fig. 4D, G). The median eye shares the same appearance as the lateral eye lobes, with a reddish outer edge and a dark inner mass (Supplementary Fig. 4F). The maximum diameter of the median eye is about 0.6 mm (40% of that of the lateral eye lobe). No evidence for the proximal region of the median eye is observed in the specimens studied.

#### Frontal appendages

Details of the frontal appendages are visible in six specimens. Micro-CT data from two further specimens confirm their presence beneath the carapace and reveal their morphology with greater clarity (SJZ-B10-753A and JS-274A, Fig. 1C, G). Frontal appendages are posterior to the lateral eye stalks and composed of 12 podomeres, divided into a base of three podomeres (Bp1 to Bp3) (Figs. 2E, F; 3B, D; 4D, F) and a claw of nine podomeres (Cp1 to Cp9) (Figs. 1C; 3B, D). Podomeres gradually taper distally (Figs. 1C and 3B). The angle at the dorsal margin between the base and claw ranges from 110° to 160°. Base podomeres are sub-trapezoidal in outline and approximately 0.6 mm long in specimen SUN 0076B (appendage about 1.7 mm long in total) (Fig. 4D, F). Their maximum height (maximum distance between dorsal and ventral margins) tends to increase toward the proximal podomere, with Bp1 having a height of about 0.6 mm, Bp2 about 0.7 mm, and Bp3 about 0.8 mm (Fig. 4D, F). The claw region appears curved inwards and dorsally in three specimens preserved in lateral aspect (Figs. 1C; 2E; 3F) but less curved in the other three specimens (one lateral view, two dorsal views) (Figs. 1D; 3A; 4A). Claw podomeres were long and rectangular (podomere length: height ratio is *c*. 1.6:1 in Cp2, and *c*. 1.5:1 in Cp8), and the overall size of individual podomeres gradually decreases distally (Figs. 1C and 3B). Podomeres of the claw limb bear paired endites, as shown on Cp1-Cp2 (micro-CT; SJZ-B10-753A) with En2 length equalling Cp2 height (Fig. 1D), and on Cp3-Cp4 (SUN 0073 A; ventral protrusion) (Fig. 3C). Cp7-Cp9 podomeres bear only a single endite (Fig. 3B, D). En5-En6 (SUN 0076B) are preserved but lack detail (Fig. 4C, E). A terminal spine on the distal-most claw podomere can be seen in both specimens that were micro-CT scanned (Fig. 1C, G) and also poorly preserved in SUN0073A (Fig. 3B, D).

#### Trunk limbs

The flap appendages were described by Zhang and Shu^23^ (based on 20 specimens) as being imbricated along the lateral sides of the trunk, each with a distally broadening, sub-ovoid outline (Fig. 2). However, their exact number was undetermined^23^, as they overlap laterally in the single specimen where they were visible. The new specimen SUN 0090, which preserves almost the whole trunk, shows 11 flap-like branches arranged along the left side of the body, one per segment, except the 8th segment with the left flap not preserved (Fig. 2C, D).

A series of eight pairs of stenopodous branches was revealed by Micro-CT scans of SJZ-B10-753A and JS-274A (Fig. 1C, G). These branches attach to the body close to the base of the flaplike branches (where they are preserved), and are segmented with at least seven podomeres (Fig. 1C). The 8th stenopodous branch projects from the underside of the trunk segment of SUN 0081 A; however, the proximal region (at least the 1st–3rd podomeres) is not present (Supplementary Fig. 4G, H). The (presumed) 4th–6th podomeres are subrectangular and taper distally, while the distal-most (presumed 7th) podomere appears as a single claw, being half the width of the 6th one (Supplementary Fig. 4G, H). Specimen JS-274A shows six flaps, each overhanging one of the 3rd to 8th stenopodous branches (Fig. 1G), but articulation between flap-like and stenopodous branches is not observed. Accordingly, the paired appendages of the 1st–8th trunk segments (thorax) are considered to consist of a flap-like branch and a stenopodous branch with at least seven podomeres.

Appendages of the 9th–11th trunk segments (abdomen) have been observed in four specimens (including three new specimens), consisting of the flap-shaped branches (Fig. 2; Supplementary Fig. 4A, B). The thoracic stenopodous branch is not observed in these specimens.

#### Preservation

Of the 52 specimens studied, 39 were preserved laterally compressed with carapace valves parallel to bedding (e.g. Fig. 1A). In contrast, the remainder were preserved dorsoventrally compressed (e.g. Fig. 1E). Among the lateral view specimens, 17 show the carapace was rotated nearly perpendicular to soft tissues (e.g. Figs. 2C; 3A; Supplementary Fig. 3A), and 8 show soft tissue protruding from the anterior and posterior margins of the carapace (e.g. Figs. 1A and 2A). In dorsal view specimens, only one specimen (SUN 0076 A/B) shows the soft body rotated nearly 270° relative to the carapace (Fig. 4A, B), and the other nine specimens show the soft parts projecting from the anterior and posterior edges of the carapace (e.g. Supplementary Fig. 3C).

### Results of phylogenetic analyses and ancestral state reconstructions

**Fig. 5:**
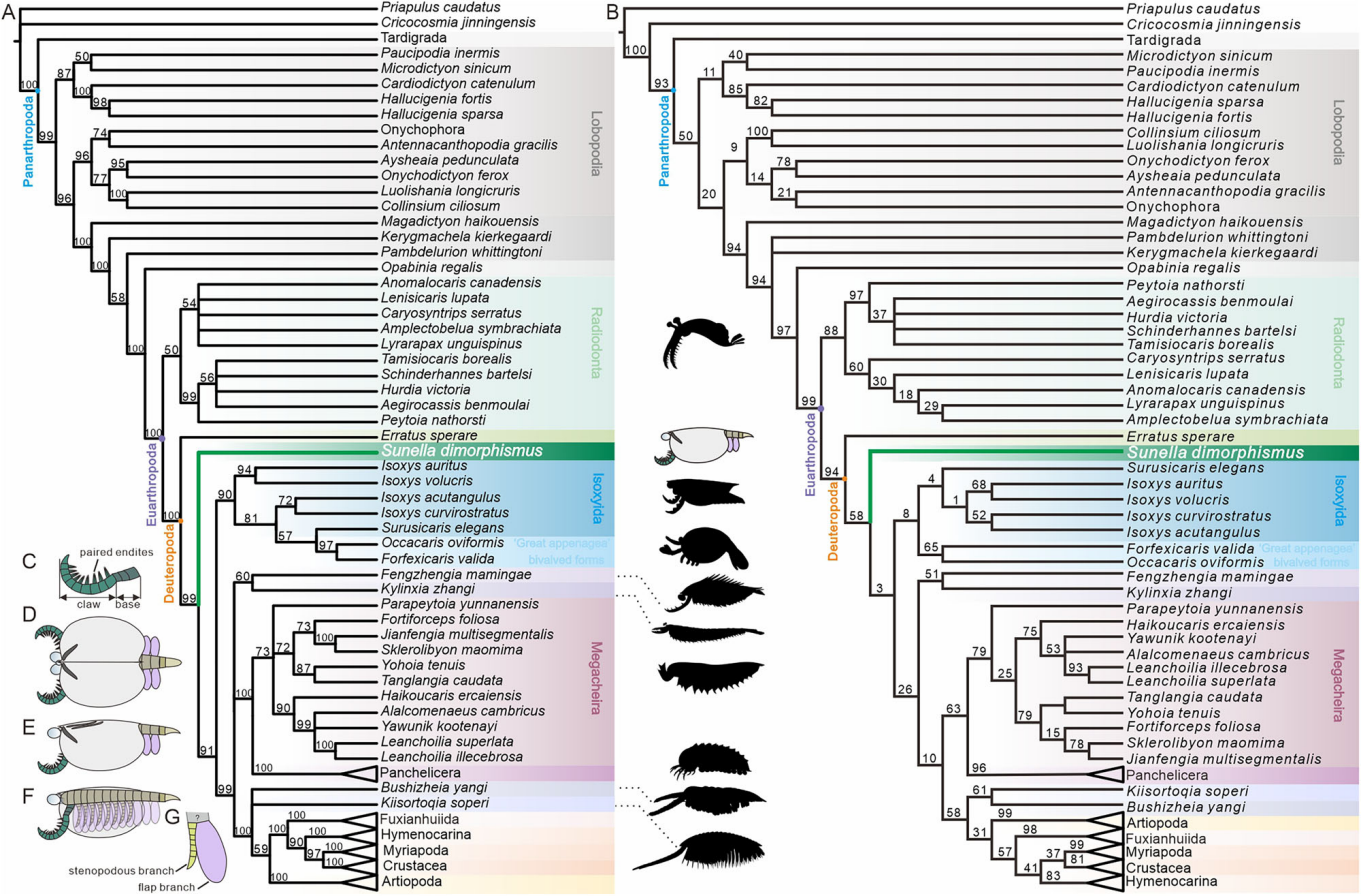
Results of cladistic analyses and interpretative diagram of *Sunella dimorphismus.* **A** simplified cladogram of *S. dimorphismus* based on results obtained by Bayesian (more details in Supplementary Fig. 5), showing *S. dimorphismus* as the earliest diverging deuteropod except *Erratus*. Nodal supports are posterior probabilities. Schematic diagram cited from https://www.phylopic.org. **B** simplified strict consensus of maximum parsimony under equal weights (more details in Supplementary Fig. 6A). Node support values from bootstrap resampling are indicated along the branches. **C**–**G** interpretative diagram of the dorsal view **D** and left view (**E**, **F**), respectively; **D**, **E** showing the dimorphic carapace with (**E**) or without anterodorsal corrugations (**D**); **F** showing the details of appendages, with **C**, **G** magnified views of the raptorial appendages and trunk appendages.

Phylogenetic analyses performed using Bayesian inference and maximum parsimony recover a grade of euarthropods with bivalved carapaces at the base of Deuteropoda, with *Erratus* the earliest diverging taxon, followed by *Sunella*, and then a clade comprising isoxyiids, *Forfexicaris*, and *Occacaris* (Fig. 5A, B; Supplementary Fig. 5A; 6A). In the implied-weights parsimony analysis, *Sunella* shifts crownward between isoxyiids and an *Occacaris*+*Forfexicaris* clade. This alternative placement, however, receives only weak support (e.g. 25 at *k* = 5, 14 at *k* = 10; Supplementary Fig. 6C, D), indicating limited topological robustness. In line with previous analyses using the same base matrix^9,10,14,15,29,30^, and many other studies^9–13,16,17,19,31,32^, Radiodonta is the earliest diverging member of the Euarthropoda^3,32^, and megacheirans are sister to chelicerates, and artiopodans to mandibulates + fuxianhuiids (Fig. 5A, B). Bayesian analysis recovers (Fig. 5A; Supplementary Fig. 5A) *Occacaris* and *Forfexicaris* within the isoxyiid clade (Fig. 5A) rather than as sister to isoxyiids as in previous versions of the matrix^9,10^ and in the equal-weights parsimony results of this study (Fig. 5B; Supplementary Fig. 6A). Importantly, *Kylinxia* and/or *Fengzhengia* are not recovered as the earliest diverging deuteropod(s), unlike previous studies using this matrix (Table 1). Instead, they are either the earliest diverging members (equal-weights parsimony) (Fig. 5B) or as sister taxa in a polytomy (Bayesian inference) (Fig. 5A) with two clades: chelicerates + megacheirans and artiopodans + fuxianhuiids + mandibulates. *Parapeytoia* was recovered within Megacheira, as in analyses using other matrices^33^.

**Fig. 6:**
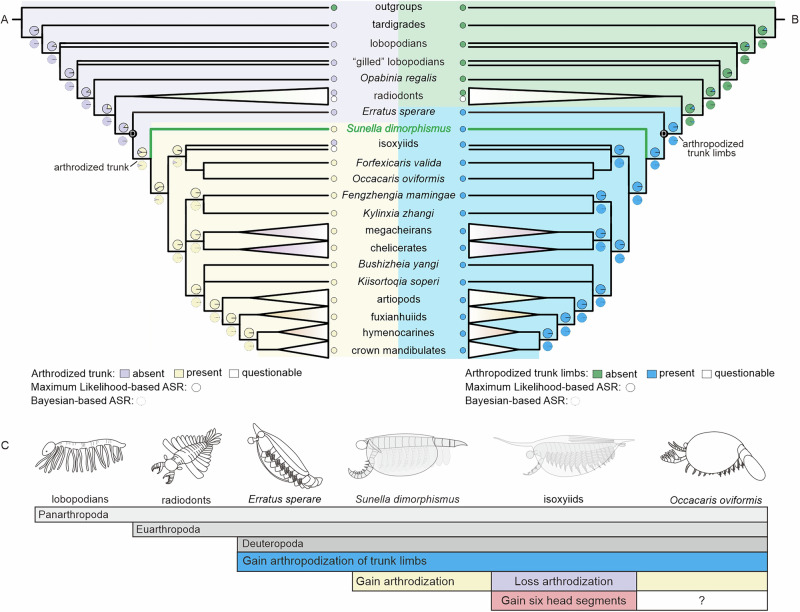
Results of ancestral state reconstructions. **A** Maximum Likelihood- and Bayesian-based ASR recovering the common ancestor of S*unella* and all other deuteropods except *Erratus* with the arthrodized trunk (details in Supplementary Figs. 7; 8). **B** Maximum Likelihood- and Bayesian-based ASR showing the common ancestor of all deuteropods with arthropodized trunk limbs (details in Supplementary Figs. 7; 8). **C** Diagrams on the representative taxa showing that trunk limb arthropodization evolved prior to arthrodization, both of which arose prior to a six-segmented head. **D** Total group of deuteropods.

Ancestral state reconstructions were conducted based on the Maximum Likelihood and Bayesian methods. Both results recover the common ancestor of *Sunella* and all other deuteropods except *Erratus* with both an arthrodized trunk and arthropodized trunk limbs (Fig. 6; Supplementary Figs. 7; 8), and the common ancestor of all deuteropods having arthropodized trunk limbs (Fig. 6; Supplementary Figs. 7; 8).

## Discussion

### The origin of arthropodized trunk appendages, arthrodized trunk, and a six-segmented head

Our results, combined with the previous studies derived from distinct base matrices^13,17^ that include both *Erratus* and *Kylinxia* as terminals in the analyses identify *Erratus* as the earliest diverging deuteropod (Table 1). All support a grade or clade of bivalved euarthropods with a carapace covering 75–100% of the body region, a pair of arthropodized, dorsally curving frontal appendages followed by a series of arthropodized biramous appendages as a more crownward-group than *Erratus*. This suggests that these matrices converge on *Erratus sperare* as the earliest diverging deuteropod, with *Kylinxia* instead placed stemwards of megacheirans, helping to resolve the sequence of key character acquisitions within the Euarthropoda.

The phylogenetic position of *Sunella* recovered in our analyses, sister to all deuteropods except for *Erratus*, refines our understanding of the sequence of character acquisition in deuteropods, in particular arthropodization, and arthrodization of the trunk, and the potential formation of a six-segmented functional head, all of which were suggested to have been present in the earliest diverging deuteropod^9,10^. Previous studies that recovered *Erratus* as the earliest diverging deuteropod—with a clade of isoxyiids as the next earliest diverging group—inferred an evolution of arthropodized trunk limbs before a fully arthrodized trunk^12^. Our ancestral state reconstructions and phylogenetic analyses concur with the inference, albeit with only a small majority of the sample following ancestral state reconstruction (Fig. 6A, B). *Erratus* records the earliest appearance of biramous trunk appendages as flaps associated with weakly sclerotized limbs^12^. *Sunella* provides the earliest acquisition of an arthrodized trunk. Accordingly, we infer that a biramous limb evolved prior to a fully arthrodized trunk, and was more simple than previous studies inferred^12^, comprising a simple flap-like exopod and stenopodous endopod as seen in *Sunella* and *Erratus*, rather than setiose, morphologically and functionally specialised limbs of *Isoxys*^12^.

The presence of segmentation in the trunk of *Sunella*, alongside the phylogenetic position of *Forfexicaris* and *Occacaris* as a clade sister to isoxyiids, suggests that the absence of trunk arthrodization in isoxyiids represents a secondary loss. This inference is supported by ancestral state reconstructions (Fig. 6A). A loss of trunk arthrodization is also known in a range of crown-group euarthropods, including an anomuran decapod *Pylopaguropsis rahayuae*^34^ and an acariform mite *Demodex*^35^, amongst others.

Micro-CT scanning reveals the alignment of head segments in *Sunella* bearing stalked eyes and raptorial appendages. This implies the discrepancies in head segmentation observed between the early branching deuteropods. A hypothesis, summarised by O’Flynn et al^10,15^., posits a six-segmented head of *Isoxys curvirostratus*. These include an ocular segment, raptorial appendages, and four short, endite-bearing biramous appendages aligned with their respective segments. Morphological reconstruction indicates a function differentiation in it’s the most anterior region, although direct neurological evidence is lacking. Thus, the acquisition of a six-segmented functional head after arthrodization and arthropodization (Fig. 6C), in the common ancestor of a clade comprising isoxyiids + *Forfexicaris* + *Occacaris* and a second clade with the remaining deuteropods. Such a six-segmented head has also been documented in crownward representatives, including *Kylinxia*^10^, *Bushizheia yangi*^15^, the megacheiran *Leanchoilia illecebrosa*^36^, the artiopodans *Sinoburius lunaris*^37^ and *Pygmaclypeatus daziensis*^38^, as well as in extant mandibulates^39^. In contrast, megacheirans such as *Fortiforceps foliosa*^33^ and *Yawunik kootenayi*^40^ have been interpreted as possessing a five-segmented head. Together with our new data from *Sunella*, these findings suggest that head segmentation is not conserved within the Deuteropoda and that a six-segmented head may have evolved convergently across different lineages.

Matrices including *Parapeytoia*^11,41^ (Table 1) have not reflected the homology scheme of ref. ^6^, and so this hypothesis remains untested by phylogenetic analyses. However, it is notable that *Isoxys* and other small bivalved euarthropods were not considered in detail by the scenario proposing *Parapeytoia* as sister to all other euarthropods (except radiodonts)^6^, and in the matrices where both *Isoxys* and *Parapeytoia* are included, *Erratus* (in this study) or *Isoxys* (with *Surusicaris*)^11^ is recovered as the earliest diverging group of deuteropods. Thus, future work interrogating the persistence of a protocerebral brain into the crown-group of Euarthropoda needs to integrate bivalved euarthropods such as *Erratus*, isoxyiids, and *Sunella*, which display unique character combinations supporting a position sister to all other deuteropods.

### Divergence of the frontal appendages in bivalved euarthropods

**Fig. 7:**
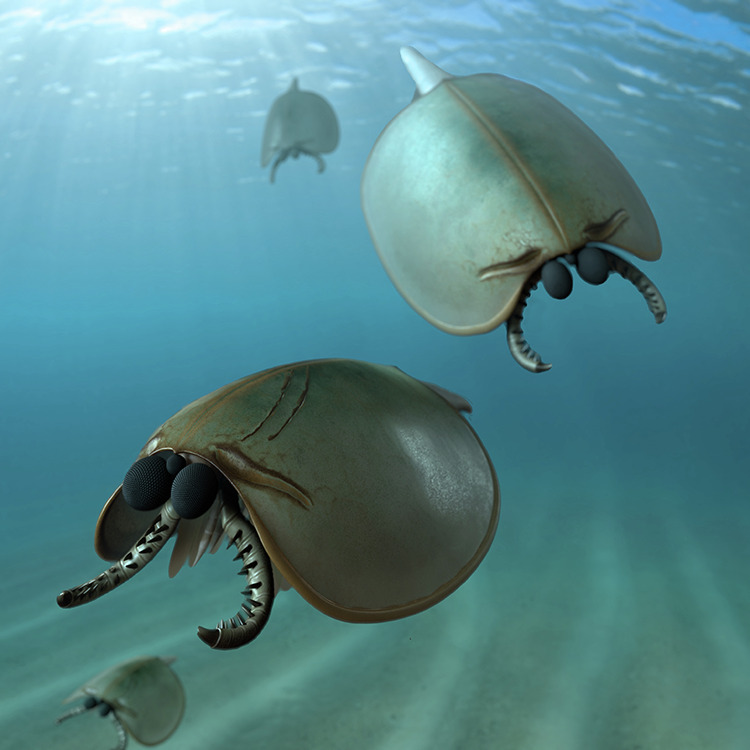
Artistic reconstruction. *Sunella dimorphismus* from the early Cambrian (Stage 3) Chengjiang biota in South China.

The frontal appendages of *Sunella* (Figs. 5C and 7) share some morphological similarities with those of radiodonts^8,31^ (Supplementary Fig. 9A), specifically in terms of their base and claw arrangement. In both taxa, the frontal appendage bears an elongate and tapering outline, and a multi-podomerous architecture that can be separated into a three-segmented proximal shaft (known in *Amplectobelua symbrachiata*^42^ and *Sunella)* and a long distal claw with paired endites (Figs. 1C, D; 2E; 3; 4). This organisation is readily distinguishable from the frontal appendages of other bivalved euarthropods. For instance, frontal appendages of *I. curvirostratus* and *Surusicaris elegans* lack a clear base region and have fewer podomeres (six and five, respectively)^13,43^. *Occacaris* and *Forfexicaris*, typical bivalved euarthropods from the Chengjiang biota, exhibit megacheiran-like frontal appendages with a prominent elbow joint, and distal podomeres with long endites^44^. In addition, many other bivalved representatives, such as *Isoxys auritus*^45^ and more crownward groups (e.g. Cambrian hymenocarines^46^ and the living mandibulates with bivalved carapace^35^) share the antenniform frontal appendages with a slender outline. It is evident that the frontal appendages are morphologically diverse, with radiodont-like, megacheiran-like, and antenniform first appendages adopted by a range of bivalved lineages. As these appendages were likely used in feeding, these data provide an additional indication that a diversification of body plans and feeding modes had already arisen among these early diverging deuteropods (*Erratus*, sunellids, isoxyiids) during the Cambrian explosion.

### Radiodont-like frontal appendages are phylogenetically widespread and convergent in Deuteropoda

The frontal appendages of radiodonts and other crown euarthropods have been the focus of much research for their vital importance in resolving the evolution of early euarthropods^3,9,10,12,14,16,47^. Similarities between the frontal appendages of radiodonts and the most anterior appendages of diverse deuteropods (e.g. *Parapeytoia*^6^, *Kylinxia*^9,10^, isoxyiids^13^, *Fengzhengia*^14^, *Bushizheia*^15^, *Kiisortoqia*^47^, megacheirans^48^) have been marshalled in support of these taxa as an early (or the earliest) diverging euarthropods or chelicerates.

A number of differences have been recognised between frontal appendages of radiodonts and radiodont-like ‘great appendages’ of these deuteropods, including attachment below or in front of the eyes in radiodonts rather than behind the eyes in deuteropods, dorsal spines present in radiodonts but absent in deuteropods, and ventral curvature in radiodonts but dorsal curvature in deuteropods (Supplementary Fig. 9A, C, D). Our phylogenetic results support a widespread distribution of ‘great appendage’ taxa among crown-group euarthropods (including the earliest diverging members of total-group mandibulates and chelicerates), while ‘great appendages’ of any kind are unknown in the earliest diverging deuteropod, *Erratus sperare*. These ‘great appendage’ euarthropods also have different body architectures. For example, *Kylinxia* and megacheirans have a fused head shield, a multi-segmented trunk, and a flat telson^9,10,14^, while the artiopodans with ‘great appendages’ have a broad, trilobed trunk^15,30,47,49^. Thus, our results do not support continuity of the radiodont frontal appendage into deuteropods, but do support the last common ancestor of mandibulates and chelicerates possessing a deutocerebral great appendage.

Under parsimony of the morphology of the deutocerebral ‘great appendage’, three patterns can be identified: the long, robust radiodont-type represented by *Sunella*, *Kylinxia*^9,10^, and probably isoxyiids (except *I. auritus*)^13,43^ and *Fengzhengia*^14^, the short elbowed megacheiran-type by ‘great appendage’ bivalved euarthropods^44^, and megacheirans^40,50^, and the slender, elongated antenniform by *I. auritus*^45^, and artiopod-related taxa (*Kiisortoqia*^47^, *Bushizheia*^15,49^, and *Kuamaia*^30^). These record the Cambrian diversification of the frontal appendage among the Deuteropoda. Notably, representatives featuring radiodont-like frontal appendages are distributed across a number of groups in our phylogenetic tree, where *Sunella* and isoxyiids locate in very basal positions as the early diverging of Deuteropoda, while *Fengzhengia* and *Kylinxia* occupy crownward placements having closer affinities with Megacheira and Chelicerata. Thus, we propose that such shared morphology in the frontal appendages of these deuteropods is likely to be a convergence.

As the position of the appendages is only a proxy for segmental affinity^13^, our interpretation of the great appendages of isoxyiids and *Sunella* as deutocerebral is subject to revision if palaeoneurological data are discovered in these taxa in the future. A protocerebral origin of these appendages would lend support to the hypotheses of ref. ^6^, albeit with *Erratus* and *Sunella* still occupying positions as sisters to all other euarthropods (except radiodonts).

### Functional morphology

The frontal appendages of *Sunella dimorphismus*, composed of many podomeres with spiniform endites, suggest a raptorial feeding function comparable to those of radiodonts and some isoxyiids^13,51,52^, while the small size in *Sunella* supports a microphagous rather than a macrophagous habit as in many large radiodonts from Chengjiang^42,52–55^. Their curved endites on proximal claw podomeres (at least En1 to En4) are compatible with grasping prey items, while the separate base region enabled greater mobility for the claw region of the appendage. The presence of large eyes on stalks suggests that *Sunella* had good visual acuity, perhaps to aid in the capture of prey but also possibly to facilitate the detection of its predators. Its trunk appendages with well-developed stenopodous branches and large ovoid flaps may have aided manoeuvrability, including ambulatory activity on the seabed and swimming, as has been suggested for Cambrian isoxyids, i.e. *Isoxys curvirostratus*^13^. The bivalved carapace may serve as protection for the soft tissues, as many Cambrian bivalved forms, such as *Isoxys*^43,45,56^, *Chuandianella*^57,58^ and *Waptia*^46^. As such, *S. dimorphismus* may represent a small and mobile predator in the Cambrian sea that was likely also prey for larger mobile animals, including other total group euarthropods.

### Summary

Our results support a more step-wise acquisition of key euarthropod characters than previous studies using the same base matrix^9,10,14,15^ with trunk limb arthropodization acquired before arthrodization and both before the evolution of a six-segmented functional head. This study also suggests that multiple phylogenetic matrices are converging on *Erratus* as the earliest diverging deuteropod through the accumulation of additional morphological data. Taxa possessing deutocerebral ‘great appendages’ are found spread across a number of groups within the phylogenetic tree, suggesting that such shared characteristic appendages may be convergent in the Deuteropoda.

## Methods

### Terminology

The terms used to describe *S. dimorphismus* morphology and their abbreviations have been derived from euarthropods from the Chengjiang and the Burgess Shale in previous studies^9,10,12,28^. The descriptions and terminologies of the frontal appendages followed those of radiodonts^52^. The maximum height of the valve is the vertical distance between the most convex points of the dorsal and ventral margin, and the maximum length is the horizontal distance at 2/3 of the valve height. For the measurement of podomere in the frontal appendages, the length is the maximum range between the proximal and distal ends, and the height refers to that between the dorsal and ventral margins (measurement data in the Supplementary Data 1).

### Optical imaging

All specimens were examined under stereomicroscopes. Observations were documented using a Canon EOS 5D Mark Ⅱ digital camera fitted with a Canon MP-E 65 mm 1–5x macro-lens and illuminated by an incandescent lamp. Camera lucida drawings were prepared using a Nikon SMZ 100 stereomicroscope. These drawings were then refined digitally in CorelDRAW X9. All photographs and illustrations were processed and compiled in Adobe Photoshop CC.

### Micro-computed tomography (Micro-CT)

To investigate structures potentially hidden beneath the carapace or within the matrix, five specimens were scanned using a Phoenix V Tome X M. Two of these were further analysed with high‑fidelity scanning using a Zeiss X-Radia 520 Versa, achieving pixel sizes of 20.00 μm and 19.85 μm, respectively. The resulting data were processed using Dragonfly v4.1.7 to generate three‑dimensional reconstructions of the fossils.

### Phylogenetic methods

The phylogenetic data matrix employed here is modified from previous researches^9,52^, with five additional characters (one for carapace [character 86] and four for frontal appendages [characters 190, 191, 193, 196]) (Supplementary Data 2). Seven additional extinct species, including three radiodonts (*Lenisicaris lupata*, *Caryosyntrips serratus*, *Tamisiocaris borealis*), *S. dimorphismus*, and three taxa bearing raptorial frontal appendages (*Fengzhengia mamingae*, *Bushizheia yangi*, *Kiisortoqia soperi*) were included in our analysis. The resulting data matrix included 89 taxa scored for 289 characters (Supplementary Data 2).

Phylogenetic analyses were conducted under two optimality criteria: Bayesian inference and maximum parsimony. Bayesian inference was conducted in MrBayes v3.2.6 under the Mkv+ gamma model. A run of 50,000,000 Markov chain Monte Carlo generations contained four chains, with trees were sampled every 1000 generations. A burn‑in of 25% was applied, and convergence was evaluated with Tracer v1.7.2^59^. The posterior probability of a clade was inferred from its frequency of occurrence in the sampled trees. Maximum parsimony analysis was performed in TNT 1.5 using the New Technology Search (NTS) method. Both equal and implied weighting schemes were applied, with k values set at 5 and 10 for implied weights (see details in Supplementary Fig. 6C, D). The analysis used a Driven Search with a minimum of 1000 searches; lineage trees were collapsed after each search. Sectorial Search, Ratchet, Drift, and Tree Fusing were activated prior to running. Bremer support values for the equally weighted tree were calculated in TNT 1.5 (Supplementary Fig. 6B). The resulting trees from both Bayesian and maximum parsimony analyses were visualised in FigTree 1.4.4, and a simplified diagram of the phylogenetic tree was prepared in CorelDRAW X9.

### Ancestral state reconstruction

Ancestral state reconstruction is a fundamental tool for exploring evolution as it provides estimates of otherwise unobservable processes^60^. To infer the evolutionary history of key morphological traits in panarthropods, we conducted ancestral state reconstructions (ASR) for various phylogenetic topologies by using multiple methods and models followed by ref. ^61^. Two informative characters were selected: arthropodized trunk limbs and arthrodized trunk. These characters were coded based on our full phylogenetic dataset (Supplementary Data 2) with character states depicted as ‘0’ (absence/inapplicable), ‘1’ (presence), or ‘?’ (questionable). To assess the evolutionary implications of tree topologies, we reconstructed ancestral states using the Bayesian approach. Bayesian ancestral states were reconstructed on trees obtained from BI standard strategies. Ancestral states were estimated using the MBASR (MrBayes Ancestral States with R)^62^ function implemented in the ‘ape’^63^ and ‘phytools’ R packages^64^. This method enables the probabilistic estimation of ancestral traits while accounting for uncertainty in character state transitions (Supplementary Date 4).

For Maximum Likelihood-based ASR, we employed ‘All-Rates-Different’ (ARD), in which every transition rate in each direction is permitted to assume a different rate^61,65^, which assumes bidirectional and symmetric transition rates between character states^66^. Model selection was based on the Akaike Information Criterion (AIC), where the model with the lowest AIC was preferred as it provided a better statistical fit to the data^67^ (Supplementary Date 5).

### Elliptical Fourier analysis

A total of 42 specimens with intact valves were selected for geometric morphometric analyses, including 19 specimens of *S. dimorphismus* (2 of them mentioned by ref. ^23^), 22 specimens of *S. grandis*^24–26^, and one for *S. shensiensis*^25^. For consistency, all specimens were imaged with the anterior end facing right; any specimens oriented to the left were mirrored. The resulting images were rendered as black silhouettes on a white background in Inkscape 1.3.2 (www.inkscape.org) and then imported into the R environment (R Core Team 2024) via the *Momocs* package^68^. The *Momocs* package was used for all Elliptical Fourier analyses and downstream analyses. All silhouettes were scaled, sampled to 64 points, and centred, and then subjected to EFA using the number of harmonics that recovered 99.9% of the total harmonic signal in the data. Results of the EFA were subjected to principal component analysis (PCA) for visualisation, and linear discriminant analysis (LDA) to determine if there were differences between the two groups (*dimorphismus* and *grandis*) (Supplementary Date 6).

### Statistics and reproducibility

No formal statistical tests were applied, as this study is based on qualitative morphological observations, micro-CT scanning, and comparative anatomical analyses of fossil specimens. Phylogenetic analyses were conducted using Bayesian inference and maximum parsimony methods. All analyses were performed using the same character matrix to ensure reproducibility. Ancestral state reconstructions were carried out using MBASR (MrBayes Ancestral States) and maximum-likelihood analyses under the all-rates-different (ARD) model. Elliptical Fourier analysis was conducted on 42 specimens using the R package *Momocs*. Raw observational data, character codings, and analytical files have been deposited in Figshare and are available upon reasonable request.

Thirty new specimens were sampled from five localities of the Chengjiang biota, i.e. the Chengjiang, Dapoutou, Jianshan, Sanjiezi, and Tanglipo (CJ, DPT, JS, SJZ and TLP), and were analyzed in this study. All these specimens have been deposited in the Shaanxi Key Laboratory of Early Life and Environments, Northwest University, Xi’an. Each specimen has been catalogued a number to facilitate independent re-examination.

### Reporting summary

Further information on research design is available in the Nature Portfolio Reporting Summary linked to this article.

## Supplementary information

Supplementary Information

Description of Additional Supplementary Files

Supplementary Data 1

Supplementary Data 2

Supplementary Data 3

Supplementary Data 4

Supplementary Data 5

Supplementary Data 6

Reporting Summary

## Data Availability

All source data in the paper are available in Figshare at https://figshare.com/s/12614553906408fe1a64^69^. The source data behind the description of carapace are available in Supplementary Data 1. The source data behind the Fig. 5 and Supplementary Figs. 5A and 6 are contained within Supplementary Data 2, while the data for the Supplementary Fig. 5B are compiled in Supplementary Data 3. The source data for the Fig. 6 and Supplementary Fig. 8 are available in Supplementary Data 4. The source data for the Supplementary Fig. 7 are available in Supplementary Data 5. The source data for the Supplementary Fig. 2A, B are available in Supplementary Data 6. Any further data required for reanalysis are available from the corresponding authors upon reasonable request.

## Code availability

The measurement data for *Sunella* valves is included as EXCEL formatted file (Supplementary Data 1). The phylogenetic datasets necessary to run the MrBayes analyses, are included as EXCEL formatted file (Supplementary Data 2). The datasets necessary to run the convergence of Bayesian analysis are included as P formatted files (Supplementary Data 3). The datasets and the commands necessary to run the Ancestral state reconstruction analyses, are included as R formatted files (Supplementary Data 4, 5). The datasets and the commands necessary to run the Elliptical fourier analyses and linear discriminant analysis, are included as R formatted file (Supplementary Data 6). The outline of the *Sunella* valve from EFA analysis is provided as a JPG formatted file (Supplementary Data 6).
