# Supplementary material for: 3D morphology of the Cambrian bivalved arthropod Sunella informs about head segmentation, arthrodization, and arthropodization
Source: Commun Biol. 2026 Mar 21;9:647. doi: 10.1038/s42003-026-09909-z (PMC13172534; doi:10.1038/s42003-026-09909-z)
Supplement: Supplementary file 1 — Supplementary Information [file 42003_2026_9909_MOESM1_ESM.pdf]

3D morphology of the Cambrian bivalved arthropod *Sunella* informs about head segmentation, arthrodization, and arthropodization

Cong Liu, Stephen Pates, Mingjing Zhang, Yu Wu, Jiaxin Ma, Dongjing Fu, and Xingliang Zhang

CONTENT

REMARKS

Derivation of name

Comparisons between *Sunella* species

Comparisons of *S. dimorphismus* with bradoriids and isoxyiids

OUTLINE ANALYSIS

SUPPLEMENTARY FIGURE

SUPPLEMENTARY REFERENCES

REMARKS

Derivation of name. The species name *dimorphismus* is after the dimorphic carapace (Supplementary Figure 1).

Comparisons between *Sunella* species.

Sunella grandis Huo, 1965, the type species of the genus, is known from the Guojiaba Formation, South Shaanxi province[1, 2], and the Shuijingtuo Formation, Hubei province[3], China. *Sunella dimorphismus* shares a similar valve size range with the type species, up to 16 mm long in adults. Unlike the carapace of *S. grandis,* which

bears long anterior and posterior cardinal spines and a broad doublure (Supplementary Figure 2C), the carapace of *S. dimorphismus* has short cardinal spines and a narrow doublure (Supplementary Figure 2D). In addition, the valve of *S. dimorphismus* exhibits an elongate outline with a greater length/height ratio than *S. grandis* (1.70 vs 1.60), and larger antero- and posterodorsal margin angles (anterior angle: 123° vs 96°; posterior angle: 117° vs 100°), as well as a larger angle between the sulcus and the hinge line (63° vs 52.5°) (Supplementary Data 1). Elliptical Fourier analysis on carapace outlines of *S. dimorphismus* and *S. grandis* supports identification of two distinct species. When Fourier coefficients are visualized using principal component analysis (PCA) two non-overlapping regions were recovered (Supplementary Figure 2A), with a Linear Discriminant Analysis (Supplementary Figure 2B) separating the two species along the single axis (Further details in section: Outline analysis below).

Sunella shensiensis was described from the Guojiaba Formation[1, 2] based on a single incomplete specimen (Hou, 1965[1], plate II 3 and 4) with a shorter cardinal spine than the type species *S. grandis* [1, 2]. Reexamination suggests *S. shensiensis* is a junior subjective synonym of *S. grandis* for the amplete lateral outline, broad doublure, and similar measurements of the length/height ratio of valve (ca. 1.60) and the angle between sulcus and hinge line (ca. 52°). Furthermore, the EFA result shows no variations in the shape of the valves between *S. shensiensis* and *S. grandis* but a difference in their shape from that of *S. dimorphismus* (Supplementary Figure 2A). Thus, we assign the single specimen of *S. shensiensis* to *S. grandis*.

Zhang and Shu reported more than 100 *Sunella* specimens from the Chengjiang

biota[4], and separated them from the *S. grandis*[1, 2] by their more elongated lateral outline and relatively short cardinal spines. The carapace of their specimens was considered to be similar to the type specimen of *S. shensiensis*, sharing features such as relatively short cardinal spines and antero- and posterodorsal curvature. Thus, Zhang and Shu referred to their specimens as *Sunella* cf. *shensiensis,* and demonstrated an additional feature, the presence of the dimorphic carapace[1]. However, the described valves of *S.* cf. *shensiensis* exhibit a greater length-to-height ratio than that of the only known specimen of *S. shensiensis* valve (1.77[4] vs. 1.64) (Supplementary Data 1), and hence a more elongated outline. Our geometric morphological results revealed that the distribution of the valve morphology of *S.* cf. *shensiensis* is distinct from that of *S. shensiensis* which overlapped with that of *S. grandis* (Supplementary Figure 2A). Herein, we consider that the specimens reported as *S.* cf. *shensiensis* by Zhang and Shu[4] represent a new species distinct from *S. grandis* and its synonym *S. shensiensis*.

In this study, the carapace of 30 new specimens of *Sunella dimorphismus* is identical to those of *S.* cf. *shensiensis* illustrated in Zhang and Shu[4]. Firstly, the valve of new specimens shares the same morphological features as *S.* cf. *shensiensis*[4], such as the subelliptical lateral outline with a convex dorsal margin, short cardinal spines accounting for about 2% of the length of the carapace, and a narrow doublure (Supplementary Figure 2D). The anterodorsal sulcus is also present in the type species *S. grandis*, which extends from the anterodorsal angle to the anteromedian part of the valve. And, remeasurement of seven of their specimens shows a similar angle

between the sulcus and the hinge line as our specimens, approximately 63° (Supplementary Data 1). Secondly, 19 new specimens show a dimorphic feature of valves with (8 specimens) or without anterodorsal corrugations (Supplementary Data 1) (Supplementary Figure 1), just like that of *S.* cf. *shensiensis*[4]. Thirdly, the measurements of 22 new specimens show a similar size range with those described as *S.* cf. *shensiensis*[4], although individuals of new specimens exhibit a larger size, ranging from 7.7 to 14.6 mm in length and 4.6 to 8.2 mm in height (Supplementary Data 1), whereas the data in Zhang and Shu[1] showed a range of valve lengths from 7.5 to 10.5 mm and heights from 4.0 to 6.0 mm. In addition, the measurements also show that the valves of 16 new specimens have an obtuse angle between the anterior and posterior margins to the dorsal edge, as do the seven specimens of *S.* cf. *shensiensis* illustrated by Zhang and Shu[4]. They both have antero- and posterodorsal margin angles of about 120°(Supplementary Data 1). Moreover, the geometric morphometric result showed no variations in valve shapes between *S. dimorphismus* and *S.* cf. *shensiensis* (Supplementary Figure 2A), indicating that they are conspecific.

A total of 25 new specimens with soft parts show that *S. dimorphismus* shares similar characters to *S.* cf. *shensiensis*[4], the possession of a pair of large lateral eyes, a single median eye, and a series of flap-like appendages arranged along the trunk. Additionally, both are known from the Yu'anshan Formation, Chengjiang biota. Thus, specimens previously described as *S.* cf. *shensiensis*[4] are here assigned to the new species *S. dimorphismus*.

Comparisons of *S. dimorphismus* with bradoriids and isoxyiids.

Previous studies have placed sunellids within bradoriids[5], an open position within the Euarthropoda[4] or, more recently, drawn comparisons between sunellids and isoxyiids[6]. Soft anatomies of *S. dimorphismus* and phylogenetic analyses (Fig. 5) allow a more comprehensive placement of sunellids in the earliest diverging deuteropods besides *Erratus*, neither within Bradoriida nor Isoxyiidae.

The carapace outline and size range of *S. dimorphismus* and other sunellids are very similar to bradoriids[7]. The CT data presented herein demonstrate that *Sunella* appendages have a distinct arrangement (more appendage pairs, lacking posteriorly directed posterior appendage pair) and morphology (flap-like lobe dorsal to likely seven-segmented stenopodous limb) to those of bradoriids – even taking into account the documented variation in bradoriid appendage morphology[8–10]. A further difference between bradoriids and *Sunella* is the presence of arthrodized segments posterior to the carapace in *Sunella,* and eyes are not known from bradoriids[10]. In summary, the new data presented here confirm that sunellids are not bradoriids.

Carapaces of sunellids, with their small anterior and posterior spines, have also been compared to isoxyiids[6]. Of isoxyiids, the overall shape of *Isoxys volucris* morphogroup A from the Sirius Passet Lagerstätte[11] most closely resembles that of *Sunella* carapaces. Both lack ornamentation and display a semicircular outline with short anterior and posterior cardinal spines. However, *Sunella* can be differentiated by its distinctive anterodorsal sulcus and a significantly narrower doublure.

The body plan of *S. dimorphismus*, which consists of a large carapace covering a multi-

segmented body, a pair of stalked lateral eyes, a pair of raptorial dorsally-curved frontal appendages, and arthropodized ventral trunk appendages, is also broadly comparable to isoxyiids[11–17]. However, *S. dimorphismus* also carries morphological features not present in *Isoxys*, including four arthrodized body segments protruding beyond the posterior margin of the carapace and the presence of a median eye. Further differences in the body organization between *Sunella* and isoxyiids include the lack of specialized appendages behind the raptorial appendages (thus a head with fewer segments than the six inferred for *Isoxys* by Ref [17]), the lack of a tail fan composed of multiple sets of flaps in *Sunella* (present in *Isoxys*), and the presence of an anterodorsal sulcus on the carapace of *Sunella* (absent in *Isoxys*). More distinctions between *Sunella* and isoxyiids can be found in the finer details of the carapace, raptorial and trunk appendages, and the number of body segments.

The raptorial appendages of *Sunella* attach behind the eyes, in a similar position to isoxyiids and deuteropods with raptorial appendages such as *Kylinxia*[18, 19]. Among members of these groups, the frontal appendages of *Sunella* are most similar to *I. volucris*, which carries a frontal appendage with at least 7 podomeres, each bearing a pair of median spinose endites[11], morphologically comparable to the claw in *S. dimorphismus*. Both taxa share a similar H/L ratio of each distal articulated podomere as well as the length/height ratio of each endite and its podomere. *Sunella* also has the same number of claw podomeres in its frontalmost appendage as *I. auritus*[15], however, the frontal appendages of *I. auritus* are antenniform with a slender outline, and podomeres are more elongate relative to their height[15]. The basal portion of the frontal

appendages has not been revealed in *I. volucris* and *I. auritus*, preventing a detailed comparison of this part. The base of the radiodont frontal appendages is inserted in front of or below the paired eyes[20, 21]. The three-segmented base – reported here in *Sunella* - is also known in amplectobeluid radiodonts[22, 23], whereas in *Kylinxia,* the base comprises only a single podomere[18]. However, while amplectobeluid and anomalocaridid radiodonts also display paired endites on podomeres of their frontal appendages[22, 24], they possess more podomeres in the claw region than *Sunella* and also have numerous dorsal spines, a feature lacking in *Sunella* altogether. *Kylinxia* frontal appendages also lack dorsal spines (as do other deuteropods with robust frontal appendages[25, 26]), however, these taxa also have more podomeres in the claw region than *Sunella.*

The trunk appendages of *Sunella* display similarities to those of *Erratus*. In particular, the presence of a flap-like exopod lacking setae and a simple stenopodous endopod lacking endites presents the interpretation that *Sunella* appendages were morphologically less differentiated and functionally less specialized than those of isoxyiids and other deuteropods. This is supported by the lack of any reduced or differentiated appendages in the head region.

As well as its smaller overall body size, *S. dimorphismus* also has fewer segments in the body (14) than isoxyiids (16 in *Surusicaris elegans* and *Isoxys curvirostratus*)[14, 16, 17] and lacks a tail fan composed of multiple flaps known in isoxyiids[11, 16], instead possessing a single projection interpreted as a caudal ramus.

OUTLINE ANALYSIS

Results

19 harmonics were retained for EFA. Principal component 1 (PC1) describes 40.2% of the variation, PC2 27.1% and PC3 16.9%. All other principal components describe 6.2% or less. The morphological variation along PC1 is from a nearly symmetric carapace with anterior and posterior spines and straight dorsal margin (negative PC1 values) to an asymmetric carapace with curved dorsal margin (positive PC1). Morphological variation along PC2 similarly shows a change from nearly symmetric carapaces with anterior and posterior spines and straight dorsal margin (negative PC2) to asymmetric carapaces with curved dorsal margin (positive PC2), however there is a posterior notch at positive PC2 shapes, and an anterior notch at positive PC1 shapes (Supplementary Figure 2A). Carapace outlines assigned to *Sunella dimorphismus* and *S. grandis* occupy distinct areas of the PC space (Supplementary Figure 2A), with the curved dorsal margin and lack of prominent spines placing *S. dimorphismus* in more positive PC1 and PC2 space than *S. grandis*. The specimen reported as *S.* cf. *shensiensis* by Zhang and Shu[4]; and specimens reported as *S. shensiensis* by Hou, 1965[1] fall within the areas of *S. dimorphismus* and *S. grandis* respectively.

Linear discriminant analysis recovers distinct clusters of *Sunella dimorphismus* and *S. grandis* along the discriminant (Supplementary Figure 2B), supported by cross-validation (92.9% correct; all 19 *S. dimorphismus* classified correctly, 20/23 *S. grandis* classified correctly).

Discussion

Sunella dimorphismus and *S. grandis* occupy two distinct areas of PC space, and can be separated using LDA into two discrete groups (Supplementary Figure 2A, B). These species differ in the shape of the dorsal margin, and prominence of the spines, as well as finer details of curvature including slight anterior and posterior notches. The clustering of *S.* cf. *sheniensis* and *S. sheniensis* specimens with *S. dimorphismus* and *S. grandis* respectively supports including specimens previously assigned to these groups to the two recognised species, *S. dimorphismus* and *S. grandis*.

For *S. dimorphismus,* carapace outline shape does not differ between the two morphs, which can be recognised from the presence of corrugations at the anterior dorsal region (Supplementary Figure 1).

SUPPLEMENTARY FIGURE

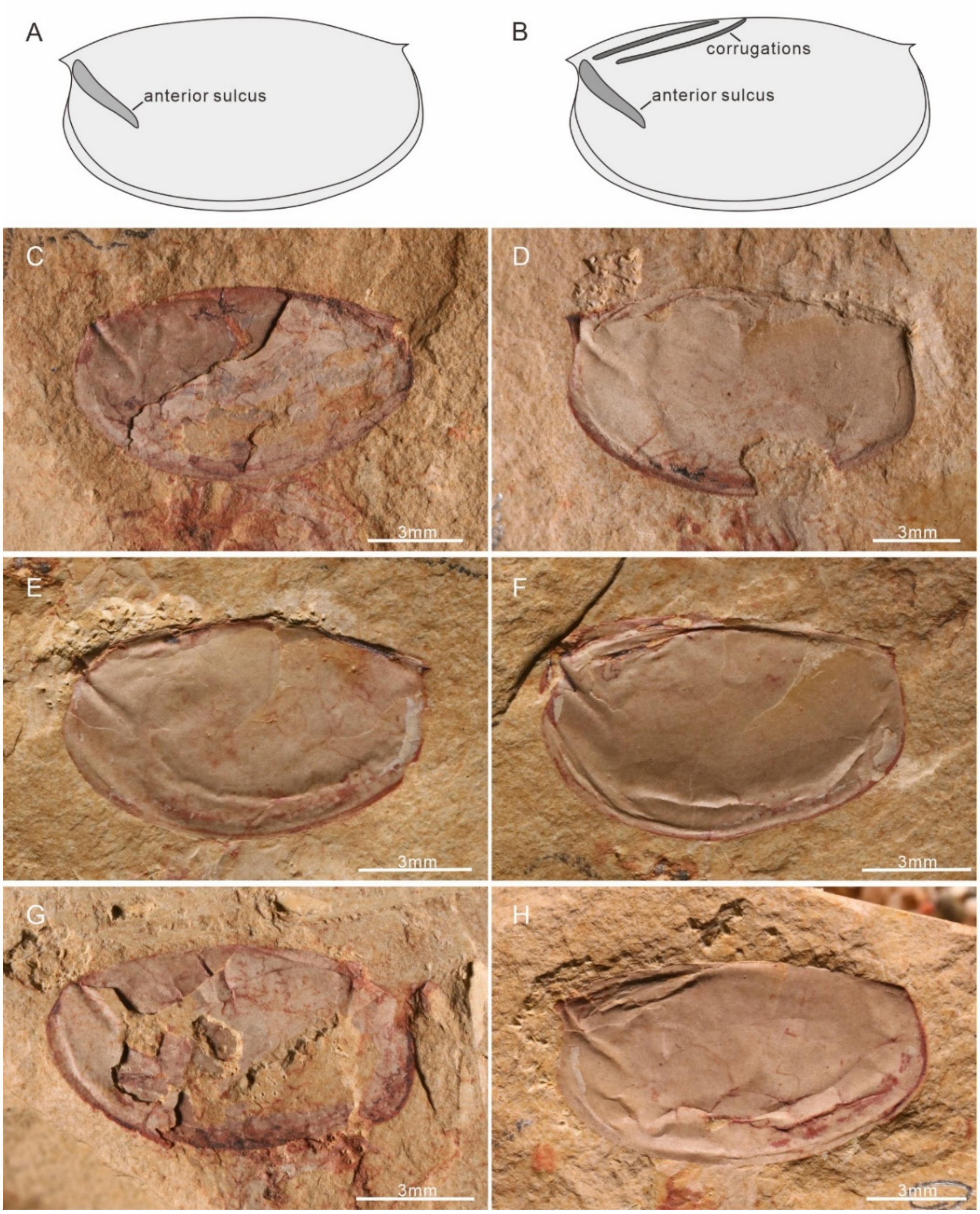

Supplementary Figure 1. Dimorphism of *Sunella dimorphismus*. A and B, schematic diagram of dimorphism valve (redrawn by Zhang and Shu[4]), showing two phenotypes: one without (C, SUN 0073A-1; E, SUN0090-2; G, SUN0100-1) and the other with two anterodorsal corrugations (D, SUN0073A-2; F, SUN0090-2; H, SUN0076A-2).

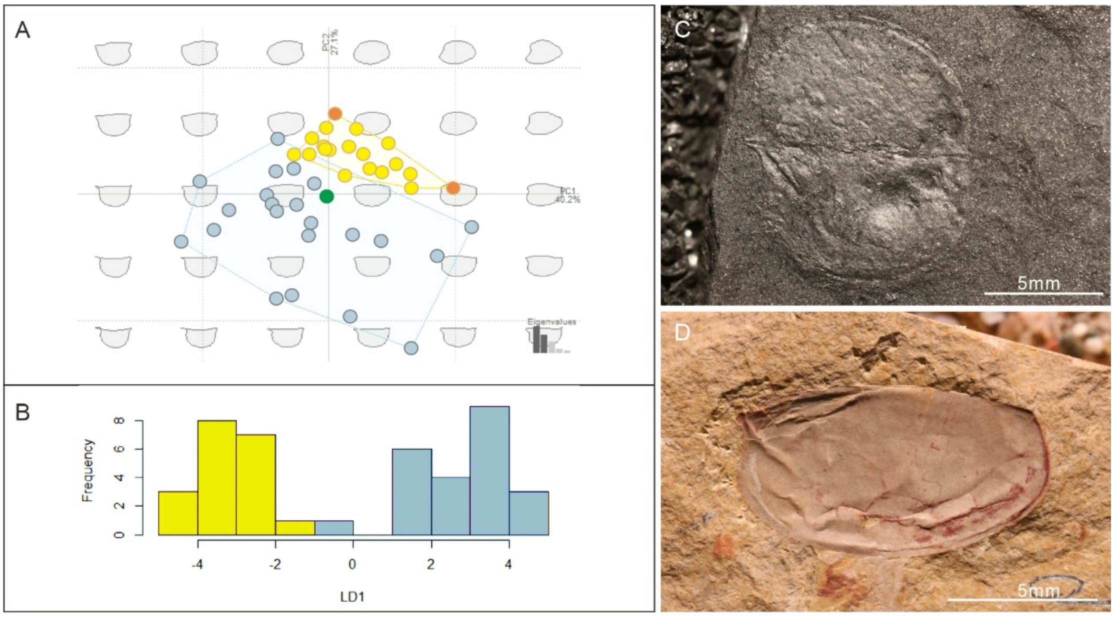

Supplementary Figure 2. Outline analysis of *Sunella* valves. A, Principal component analysis (PCA) visualizing PC1 and PC2 of result of elliptical Fourier analysis on *Sunella* carapace outline shapes. Yellow point: *S. dimorphismus*; orange point: specimen reported as *S.* cf. *shensiensis* by Zhang and Shu[4]; blue point: *S. grandis*; green point: specimen reported as *S. shensiensis* by Hou, 1965[1]. B, result of linear discriminant analysis (LDA) showing distinct clusters of *Sunella dimorphismus* and *S. grandis* along the discriminant. C and D, showing the valve outline of *S. grandis* (*C*, LELE-MX098 from the Shuijingtuo Formation in Hubei Province, China) and *S. dimorphismus* (*D*, SUN0076A-1 from the Helinpu Formation in Yunnan Province, China) respectively.

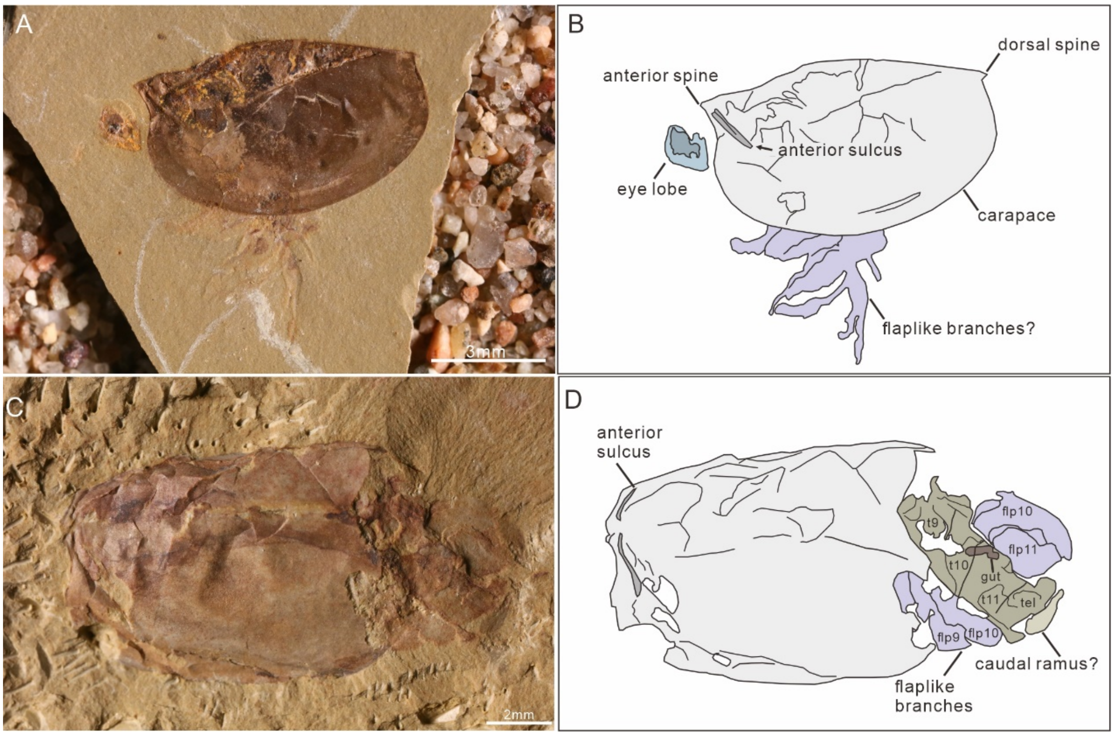

Supplementary Figure 3. ***Sunella dimorphismus*** **from the Chengjiang biota**. A and B, SJZ-B18-871, a general view of a twisted specimen, a subelliptical valve with short anterior and posterior cardinal spines, anterodorsal sulcus extended from the anterodorsal angle to the anteromedian part of the valve, rounded eye lobes consisted of a light outer layer and a dark internal mass, and flapped appendage traces. C and D, SUN 0083A, dorsal view, showing the 9th to 11th segments, a telson, and a caudal ramus projecting behind the posterior margin of the carapace, gut, and flaplike appendages.

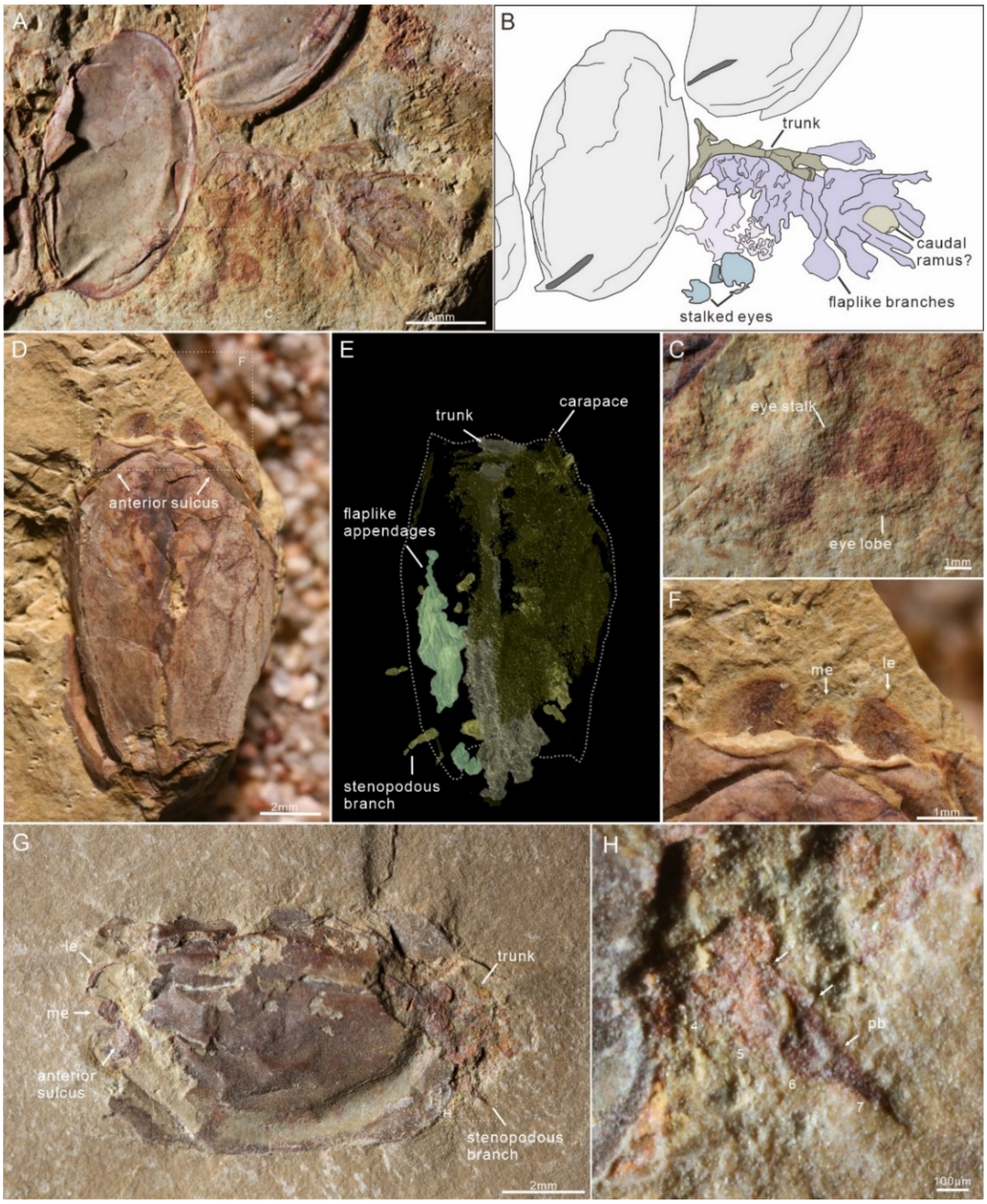

Supplementary Figure 4. Eyes and segmented trunk appendage in *Sunella dimorphismus*. A, SUN 0100, lateral view, specimen with a 90° rotated carapace, showing a pair of stalked eyes (close up in C), traces of flaplike appendages, and an ovoid, paddle-shaped ramus. B, camera-lucida drawing of *A*. D-F, SUN 0095. D, dorsal view, showing a pair of lateral eyes and a single median eye. E, rendering model, showing trunk appendages encompassing stenopodous branch and flap-like branch. G and H, SUN 0081A, dorsal view, showing segmented 8th stenopodous branch. le: left eye lobe.

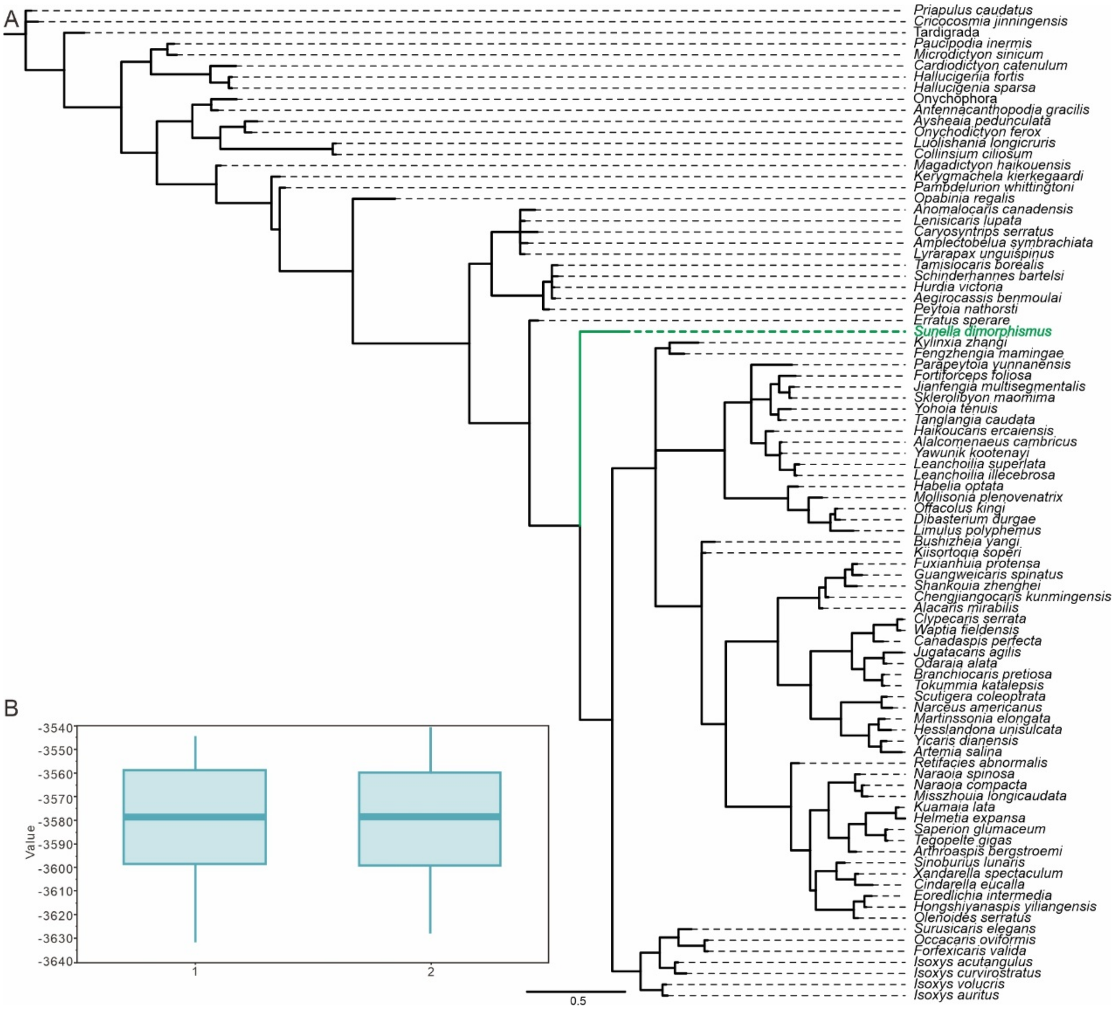

Supplementary Figure 5. Consensus tree from Bayesian phylogenetic inference analysis. A, the tree from a Bayesian analysis, showing *S. dimorphismus* (in green) as the earliest diverging deuteropods besides *Erratus*. Scale bar to indicate expected changes per site. B, box and whisker plots of the two posterior samples of Bayesian analysis, supporting a converged result.

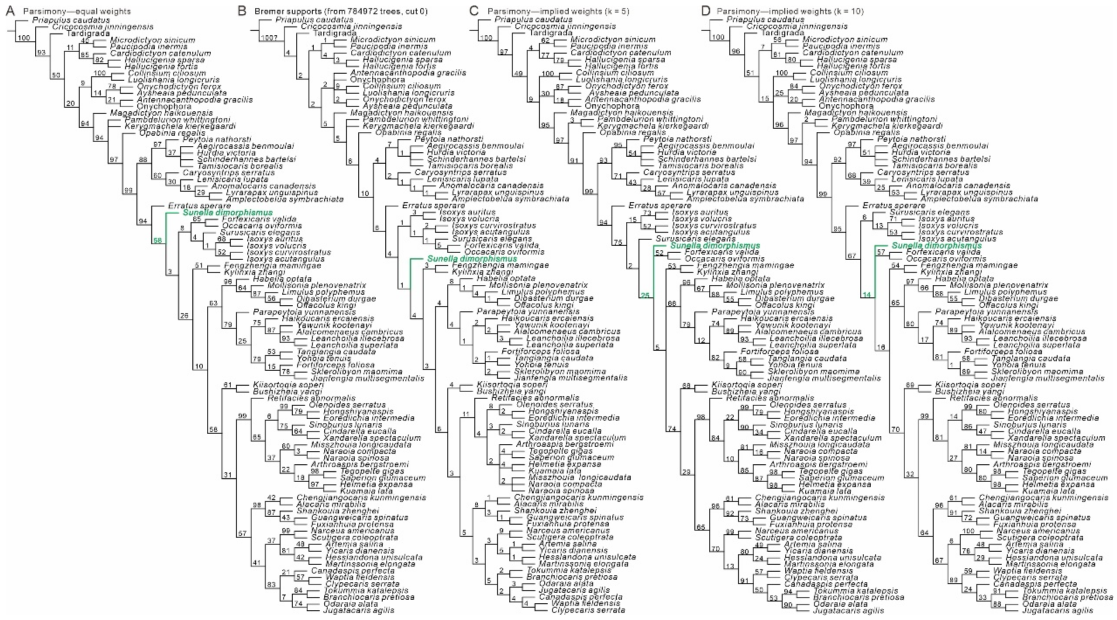

Supplementary Figure 6. Strict consensus trees from maximum parsimony. A, strict consensus tree from maximum parsimony analysis under equal weight shows *S. dimorphismus* (in green) as the earliest diverging deuteropod besides *Erratus* (node support = 58), reinforcing the reliability of this phylogenetic placement. B, the bremer support (branch support) values calculated with equal weighted parsimony tree. A value of 1 indicates weak support for a crownward placement of *S. dimorphismus* to the monophyletic group, including isoxyiids and an *Occacaris* + *Forfexicaris* clade. C and D, strict consensus tree from maximum parsimony under implied weights (*C*, k = 5 and *D*, k = 10), recovering *S. dimorphismus* between the isoxyiids and a clade containing *Occacaris* and *Forfexicaris*. The node support values for this placement are low (25 and 14, respectively; both < 50), indicating limited robustness of this phylogenetic relationship.

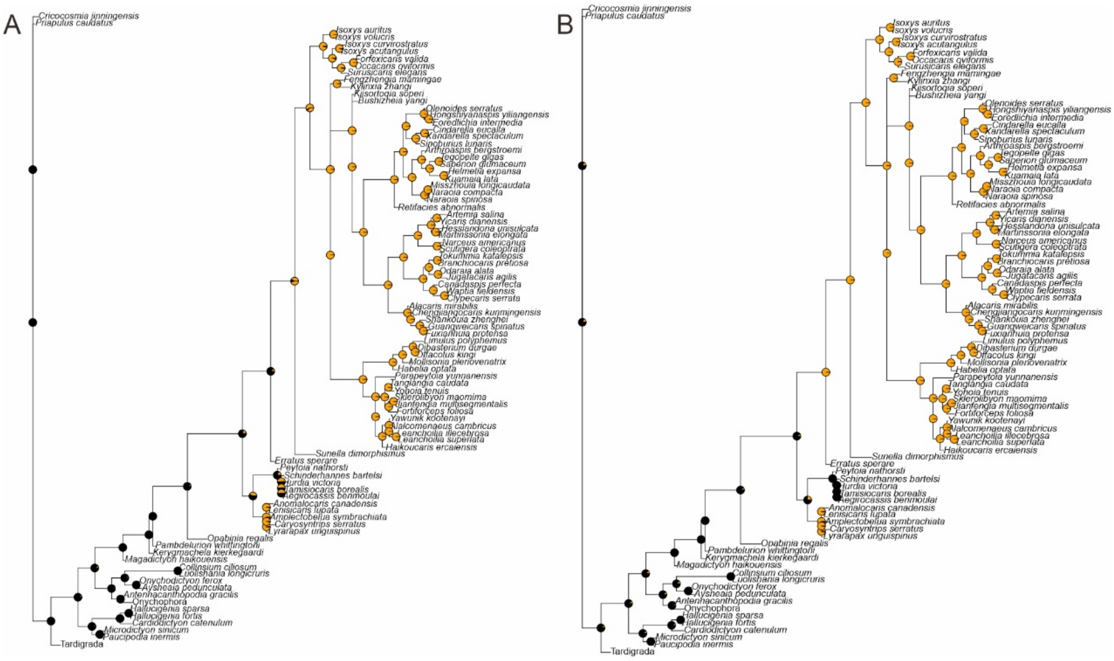

Supplementary Figure 7. Results of the Maximum Likelihood-based ancestral state reconstructions. Maximum likelihood-based ASR utilises the 'All-Rates-Different' model, showing the common ancestor of *Sunella* and all other deuteropods except *Erratus* with both an arthrodized trunk (*A*) and arthropodized trunk limbs (*B*). The common ancestor of Deuteropoda having arthropodized trunk limbs (*B*).

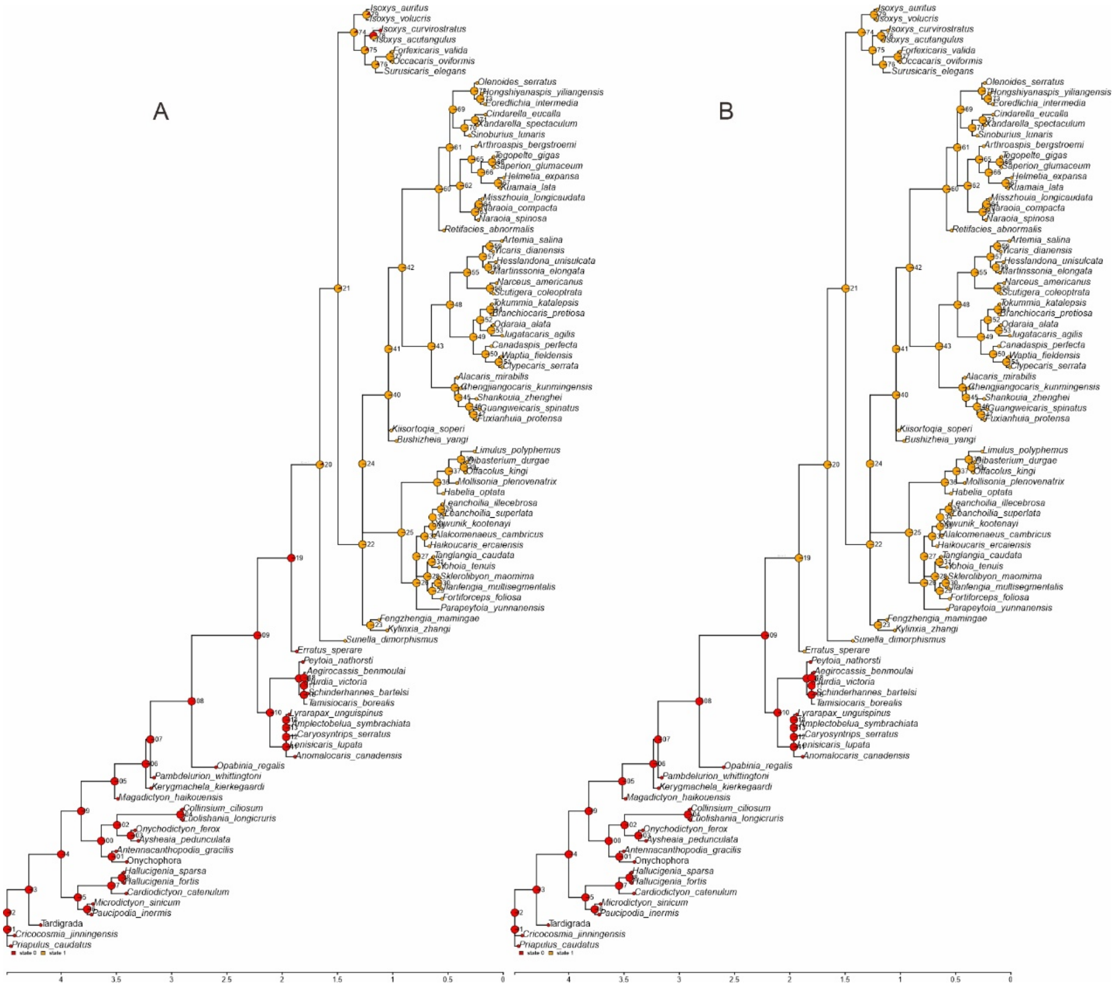

Supplementary Figure 8. Results of the Bayesian-based ancestral state reconstructions. Bayesian ancestral states estimated using the MBASR function implemented in the 'ape' and 'phytools' R packages, showing the origin of an arthrodized trunk (*A*) at the node, where *Sunella* is recovered sister to all other deuteropods (except *Erratus*), and the common ancestor of all deuteropods with arthropodized trunk limbs (*B*).

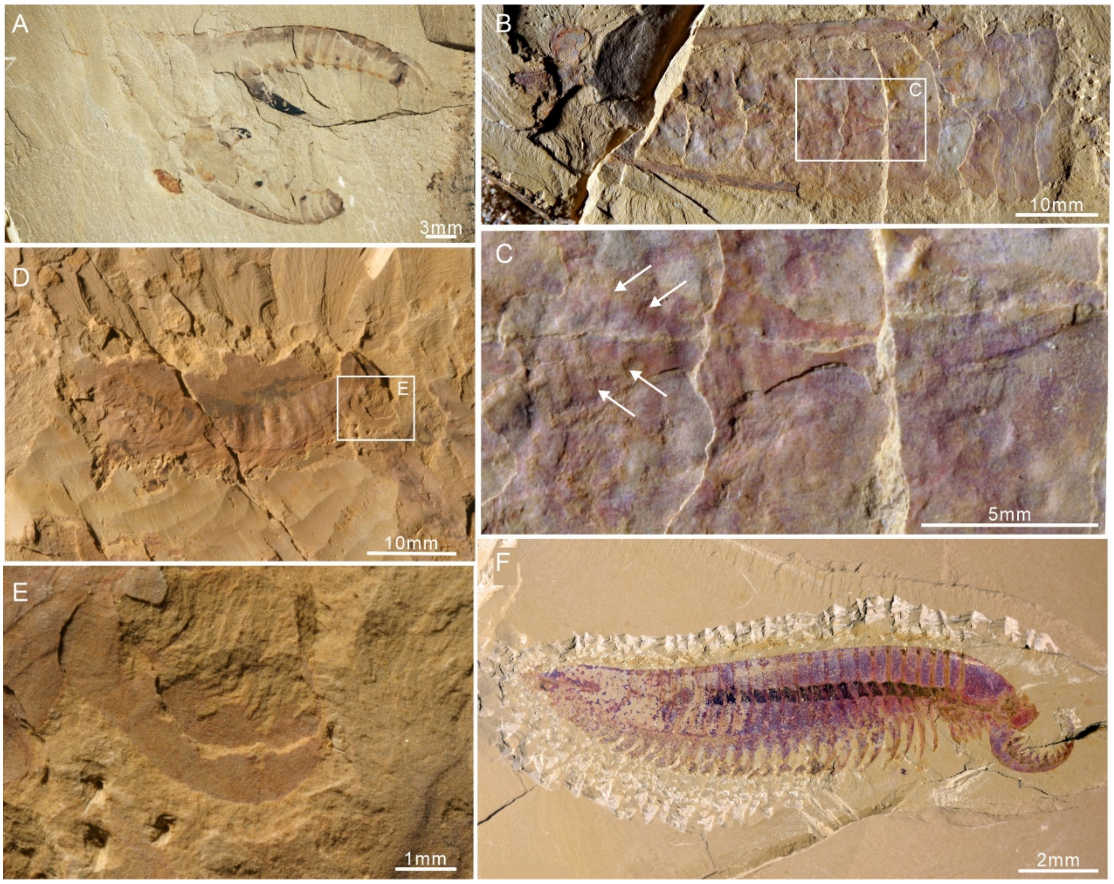

Supplementary Figure 9. Representative euarthropods from the Cambrian (Stage 3) of the Yu'anshan shale Member, Yunnan, China. A, *Amplectobelua symbrachiata* frontal appendages (EJ1889, deposited in the Shaanxi Key Laboratory of Early Life and Environments) from Erjie section. B, *Erratus sperare* (XDBZ101, deposited in the Shaanxi Key Laboratory of Early Life and Environments) from Jianshan section, showing arthropodized trunk limbs (*C*, arrows indicate the segmental boundaries of endopods). D, *Isoxys curvirostratus* (JS0014, deposited in the Shaanxi Key Laboratory of Early Life and Environments) from Jianshan section, showing a pair of frontal appendages (*E*). F, *Kylinxia zhangi* (YLSNHM 01124, deposited in Yingliang Stone Natural History Museum) from the Haikou section, showing a pair of frontal appendages with endites.

SUPPLEMENTARY REFERENCES

1. Huo, S. Additional notes on Lower Cambrian Archaeostraca from Shensi and Yunnan. *Acta Palaeontogica Sinica* **13**, 291–307 (1965). (In Chinese and English summary)
2. Huo, S. & Shu, D. *Cambrian Bradoriida of South China* (The Northwest University Press, 1985). (In Chinese with English summary)
3. Sun, A. et al. Taxonomy and ontogeny of bivalved arthropods from the lower member of the Shuijingtuo Formation, Series 2 and Stage 3, Eastern Three Georges Area, South China. *Acta Palaeontol. Sin.* **60**(1),187–199 (2021).
4. Zhang, X. & Shu, D. Soft anatomy of sunellid arthropods from the Chengjiang Lagerstätte, lower Cambrian of southwest China. *J. Paleont.* **81**(6), 1422–1432 (2007).
5. Williams, M., Siveter, D.J., Popov, L.E. & Vannier, J. Biogeography and affinities of the bradoriid arthropods: Cosmopolitan microbenthos of the Cambrian seas. *Palaeogeogr. Palaeoclimatol. Palaeoecol.* **248**, 202–232 (2007).
6. Chen, F., Betts, M.J., Zhang, Z. & Brock, G.A. The bivalved arthropod *Caudicaella* aff. *bispinata* from the Heatherdale Shale (Cambrian Stage 3), South Australia. *Palaeoworld* **34**(3): 100882 (2025).
7. Cox, A. & Pates, S. Geographic, taxonomic, and temporal interrogation of bradoriid diversity and carapace disparity. *Palaeontol. Electronica* **27**(3): a56 (2024).
8. Hou, X., Siveter, D. J., Williams, M., Walossek, D. & Bergström, J. Appendages of the arthropod *Kunmingella* from the Early Cambrian of China: Its bearing on the systematic position of the Bradoriida and the fossil record of the Ostracoda. *Philos. T. R. Soc. B* **351**, 1131–1145 (1996).

9. Hou, X., Williams, M., Siveter, D.J., Aldridge, R.J. & Sansom, R.S. Soft–part anatomy of the Early Cambrian bivalved arthropods *Kunyangella* and *Kunmingella*: significance for the phylogenetic relationships of Bradoriida. *P. Roy. Soc. B* **277**, 1835–1841 (2010).
10. Zhai, D. et al. Variation in appendages in early Cambrian bradoriids reveals a wide range of body plans in stem-euarthropods. *Commun. Biol.* **2**:329 (2019).
11. Nielsen, M.L., Rasmussen, J.A. & Harper, D.A.T. Sexual dimorphism within the stem-group arthropod *Isoxys volucris* from the Sirius Passet Lagerstätte, North Greenland. *Bull. Geol. Soc. Den.* **65**, 47–58 (2017).
12. García-Bellido, D.C. et al. The bivalved arthropods *Isoxys* and *Tuzoia* with soft-part preservation from the Lower Cambrian Emu Bay Shale Lagerstätte (Kangaroo Island, Australia). *Palaeontology* **52**, 1221–1241 (2009).
13. Stein, M., Peel, J.S., Siveter, D.J. & Williams, M. *Isoxys* (Arthropoda) with preserved soft anatomy from the Sirius Passet Lagersätte, Lower Cambrian of North Greeland. *Lethaia* **43**, 258-265 (2010).
14. Fu, D., Zhang, X. & Shu, D. Soft anatomy of the Early Cambrian arthropod *Isoxys curvirostratus* from the Chengjiang biota of South China with a discussion on the origination of great appendages. *Acta Palaeontol. Pol.* **56**, 843–852 (2011).
15. Fu, D., Zhang, X., Budd, G.E., Liu, W. & Pan, X. Ontogeny and dimorphism of *Isoxys auritus* (Arthropoda) from the early Cambrian Chengjiang biota, South China. *Gondwana Res.* **25**, 975–982 (2014).
16. Aria, C. & Caron, J.-B. Cephalic and limb anatomy of a new isoxyid from the Burgess Shale and the role of 'stem bivalved arthropods' in the disparity of the frontal most appendage. *PLoS ONE* **10**, e0124979 (2015).
17. Zhang, C. et al. Three-dimensional morphology of the biramous appendages in

Isoxys from the early Cambrian of South China, and its implications for early euarthropod evolution. *Proc. R. Soc. B* **290**: 20230335 (2023).

18. Zeng, H., Zhao, F., Niu, K, Zhu, M. & Huang, D. An early Cambrian euarthropod with radiodont-like raptorial appendages. *Nature* **588**, 101–105 (2020).
19. O'Flynn, R.J. et al. The early Cambrian *Kylinxia zhangi* and evolution of the arthropod head. *Current Biology* **33**, 1–8 (2023).
20. Cong, P., Ma, X., Hou, X., Edgecombe, G. D. & Strausfeld, N.J. Brain structure resolves the segmental affinity of anomalocaridid appendages. *Nature* **513**, 538–542 (2014).
21. Moysiuk, J. & Caron, J.-B. A three-eyed radiodont with fossilized neuroanatomy informs the origin of the arthropod head and segmentation. *Current Biol.* **32**, 3302–3316 (2022).
22. Cong, P., Daley, A.C., Edgecombe, G.D. & Hou, X. The functional head of the Cambrian radiodontan (stem-group Euarthropoda) *Amplectobelua symbrachiata*. *BMC Evol. Biol.* **17**, 208 (2017).
23. Cong, P. et al. New radiodonts with gnathobase-like structures from the Cambrian Chengjiang biota and implications for the systematics of Radiodonta. *Pap. Palaeontol.* **4**, 605–621 (2018).
24. Daley, A.C. & Edgecombe, G.D. Morphology of Anomalocaris canadensis from the Burgess Shale. *J. Paleontol.* **88**, 68–91 (2014).
25. O'Flynn, R.J. et al. A new euarthropod with 'great appendage'-like frontal head limbs from the Chengjiang Lagerstätte, Southwest China. *Palaeontol. Electronica* **23**(2): a36 (2020).
26. O'Flynn, R.J. et al. The early Cambrian *Bushizheia* yangi and head segmentation in upper stem-group euarthropods. *Pap. Palaeontol.* e1556 (2024).
