# Supplementary material for: 3D morphology of the Cambrian bivalved arthropod Sunella informs about head segmentation, arthrodization, and arthropodization
Source: Commun Biol. 2026 Mar 21;9:647. doi: 10.1038/s42003-026-09909-z (PMC13172534; doi:10.1038/s42003-026-09909-z)
Supplement: Supplementary file 2 — Description of Additional Supplementary Files [file 42003_2026_9909_MOESM2_ESM.pdf]

Description of Additional Supplementary files

File name: Supplementary Data 1

Description: Source data underlying Description of Carapace.

File name: Supplementary Data 2

Description: Character matrix used in phylogenetic analyses. Source data underlying Fig.5 and Supplementary Fig. 5A and 6.

File name: Supplementary Data 3

Description: Source data underlying Supplementary Fig. 5B.

File name: Supplementary Data 4

Description: Source data underlying Fig. 6 and Supplementary Fig. 8.

File name: Supplementary Data 5

Description: Source data underlying Fig. 6 and Supplementary Fig. 7.

File name: Supplementary Data 6

Description: Source data underlying Supplementary Fig. 2 A and B.
