# Supplementary material for: 3D morphology of the Cambrian bivalved arthropod Sunella informs about head segmentation, arthrodization, and arthropodization
Source: Commun Biol. 2026 Mar 21;9:647. doi: 10.1038/s42003-026-09909-z (PMC13172534; doi:10.1038/s42003-026-09909-z)
Supplement: Supplementary file 4 — Supplementary Data 2 [file 42003_2026_9909_MOESM4_ESM.zip › Supplementary character list.docx]

**CHARACTER LIST**

The following list is taken from the Zeng et al.^1^, and includes the modifications made for this study as well as those from Wu et al.^2^. Character headings refer to the original publication by [ZEX] and [WUX], where X is the corresponding number for that character in Zeng et al. (2020) and Wu et al. (2024), respectively.

| **Cuticle** |
| --- |

1. Annulation of integument on main body [ZE2020-1]

(0) absent

(1) present

1. External metameric boundaries on the main body [ZE2020-2]

(0) invisible or ambiguous

(1) visible and delimited

1. Sclerotization of main body [ZE2020-3]

(0) absent

(1) present

*Remark*: According to Zhang et al.^3^, we here recode *I. curvirostratus* as ‘0’, and other isoxyids as ambiguous. We recoded the arthrodization and arthropodization (trunk limbs) of the radiodonts *Anomalocaris canadensis*^4^, *Lyrarapax unguispinus*^5, 6^, *Schinderhannes bartelsi*^7^, and *Hurdia victoria*^8^ as “0”, while others as uncertain due to the lack of trunk details.

1. Arthrodization of main body [ZE2020-4]

(0) absent

(1) present

*Remark*: Same as previous.

1. Integumental sclerites or nodes on the head [ZE2020-5]

(0) absent

(1) present

1. Integumental sclerites or nodes on trunk [ZE2020-6]

(0) absent

(1) present

1. Integumental sclerites with extended reticulated plate-like base [ZE2020-7]

(0) absent

(1) present

1. Integumental sclerites with a prominent spine [ZE2020-8]

(0) absent

(1) present

1. Integumental sclerites, relative height to trunk diameter [ZE2020-9]

(0) shorter or comparable

(1) at least 1.5 times longer

1. Integumental sclerites, more than two elements per metamere [ZE2020-10]

(0) absent

(1) present

1. Integumental sclerites, paired spines per metamere [ZE2020-11]

(0) absent

(1) present

1. Integumental sclerites, size change along body axis [ZE2020-12]

(0) close

(1) anteriormost and posteriormost ones smaller, intermediate ones larger

1. Papillae or other cuticular derivatives on trunk annuli [ZE2020-13]

(0) absent

(1) present

Common in many lobopodians and Onychophora.

1. Tergites [ZE2020-14]

(0) absent

(1) present

*Remark*: We here recode *Surusicaris elegans*^9^ and *I. curvirostratus*^3^ as ‘0’, and other isoxyids as ‘?’. Code *Occacaris_oviformis* as present^10^, while *Forfexicaris valida*^11^ as ‘?’.

1. Sternites [ZE2020-15]

(0) absent

(1) present

*Remark*: Same as previous except *Occacaris oviformis*^10^ as ambiguous.

1. Pleurites [ZE2020-16]

(0) reduced or fused

(1) developed

*Remark*: Same as previous.

1. Cuticle biomineralized [ZE2020-17]

(0) absent

(1) present

| **Eyes** |
| --- |

1. Eyes [ZE2020-18]

(0) absent

(1) present

1. Ocelli as primary eyes [ZE2020-19]

(0) absent

(1) present

1. Compound eyes [ZE2020-20]

(0) absent

(1) present

1. Eye stalks [ZE2020-21]

(0) absent or very reduced

(1) present

1. Anteroposterior position of compound eyes in head [ZE2020-22]

(0) anteriormost

(1) anterior

(2) middle

(3) posterior

1. Compound eyes accommodated by dorsal bulge on fused head shield [ZE2020-23]

(0) absent,

(1) present

1. Eye stalks of compound eyes covered by fused head shield [ZE2020-24]

(0) absent

(1) present

1. Eye stalks of compound eyes incorporated into fused head shield, forming eye ridges [ZE2020-25]

(0) absent

(1) present

1. Compound eyes with eye slits [ZE2020-26]

(0) absent

(1) present

1. Compound eyes bounded by suture on fused head shield [ZE2020-27]

(0) absent

(1) present

1. Lenses calcified [ZE2020-28]

(0) absent

(1) present

1. Median eyes [ZE2020-29]

(0) absent

(1) present

1. Number of median eyes [ZE2020-30]

(0) one

(1) two

(2) three

(3) at least four

*Remark*: According to O’Flynn et al.^12^, we here recode *Kylinxia* as ‘0’.

1. Four or five eyes arranged in a sub-transverse band across head shield [ZE2020-31]

(0) absent

(1) present

*Remark*: Same as previous, we recoded ‘0’ for the *Kylinxia*.

1. Five compound eyes with size differentiation [ZE2020-32]

(0) absent

(1) present

*Remark*: Same as previous.

1. Relative diameter of compound eyes compared with length of bivalved carapace [ZE2020-33]

(0) < 5%

(1) 5–10%

(2) > 10%

| **MOUTH** |
| --- |

1. Position of mouth opening [ZE2020-34]

(0) terminal

(1) ventral

1. Orientation of mouth opening [ZE2020-35]

(0) anterior

(1) ventral

(2) posterior

1. Telescoping feeding apparatus [ZE2020-36]

(0) absent

(1) present

1. Circumoral structures in a radial arrangement [ZE2020-37]

(0) absent

(1) present

1. Differentiation of elements in the outer ring of circumoral structures [ZE2020-38]

(0) absent

(1) present

1. Number of inner spines on the elements in the outer ring of circumoral structures [ZE2020-39]

(0) absent or single

(1) multiple

1. Sclerotized circumoral plates in a radial arrangement [ZE2020-40]

(0) absent

(1) present

1. Sclerotized circumoral plates in a radial arrangement (radiodont-type oral cone) [ZE2020-41]

(0) absent

(1) present

1. Symmetrical pattern of radiodont-type oral cone [*ZE2020-42*]

(0) triradial

(1) tetraradial

1. External surface of sclerotized circumoral plates [ZE2020-43]

(0) smooth or bearing weak nodes

(1) bearing prominent scale-like nodes

1. Furrowed folds on sclerotized circumoral plates [ZE2020-44]

(0) absent

(1) present

1. Labrum [ZE2020-45]

(0) absent

(1) present

1. Hypostome [ZE2020-46]

(0) absent

(1) present

1. Hypostome attachment [ZE2020-47]

(0) wide attachment, with or without suture

(1) natant

(2) narrow overlap with pre-hypostome

(3) narrow attachment at hypostomal suture

1. Hypostome accommodating antennae and extensively covering the mouth [ZE2020-48]

(0) absent

(1) present

1. Hypostome butterfly-shaped [ZE2020-49]

(0) absent

(1) present

| **Digestive** **system** |
| --- |

1. Gut morphology [ZE2020-50]

(0) simple and straight

(1) bearing a series of digestive diverticula or glands

1. Gut, triangular lateral extension into trunk appendages [ZE2020-51]

(0) absent

(1) present

1. Branching digestive diverticula in head [ZE2020-52]

(0) absent

(1) present

1. Anus opening, position [ZE2020-53]

(0) terminal

(1) ventral

| **Body (non-appendicular structures)** |
| --- |

1. Metamerism of main body [ZE2020-54]

(0) absent

(1) present

1. Number of body metameres [ZE2020-55]

(0) ≤ 20

(1) 21–24

(2) 25–37

(3) 38–43

(4) ≥ 44

1. Number of dorsal divisions or tergites [ZE2020-56]

(0) ≤ 7

(1) 8–13

(2) 14–19

(3) 20–33

(4) ≥ 34

1. Size change of main body along body axis [ZE2020-57]

(0) cylindrical

(1) broad anteriorly and tapering posteriorly

| **Head (non-appendicular structures)** |
| --- |

1. Anterior paired projections [ZE2020-58]

(0) absent

(1) present

1. Head sclerotization [ZE2020-59]

(0) absent

(1) present

1. Unsclerotized head part possessing non-appendicular proboscis with a clear posterior boundary [ZE2020-60]

(0) absent

(1) present

1. Unsclerotized head part, anteriormost part adjoined to bases of frontalmost appendages [ZE2020-61]

(0) absent

(1) present

1. Unsclerotized head part protruded [ZE2020-62]

(0) absent

(1) present

1. Unsclerotized head part inflated, narrowed at base of protrusion [ZE2020-63]

(0) absent

(1) present

1. Unsclerotized head part elongated and tubular [ZE2020-64]

(0) absent

(1) present

1. Anteriormost sclerite associated with eyes (ocular sclerites) [ZE2020-65]

(0) absent

(1) present

1. Anteriormost sclerites forming a sclerite complex [ZE2020-66]

(0) absent

(1) present

1. Configuration of anteriormost sclerites [ZE2020-67]

(0) central element oval, lateral elements connected by peduncular structures

(1) central ‘H-element’ and paired lateral ‘P-elements’

1. Ocular sclerite as a dorsal plate with pronounced marginal rim covering anteriormost head part [ZE2020-68]

(0) absent

(1) present

1. Expanded head sclerite with posterior notches accommodating eyes, covering a large head region [ZE2020-69]

(0) absent

(1) present

1. Ocular sclerite accommodated by fused head shield [ZE2020-70]

(0) absent

(1) present

1. Ocular sclerite accommodated by bivalved head carapace [ZE2020-71]

(0) absent

(1) present

1. Ocular sclerite accommodated by semi-circular head carapace [ZE2020-72]

(0) absent

(1) present

1. Ocular sclerite accommodated by notched fused head shield [ZE2020-73]

(0) absent

(1) present

1. Ocular sclerite covered by true head shield [ZE2020-74]

(0) absent

(1) present

1. Post-ocular tergal sclerotization in head [ZE2020-75]

(0) absent

(1) present

1. Carapace connected to the head region at its anterior part, posterior part free [ZE2020-76]

(0) absent

(1) present

1. Simple head carapace covering only a few anterior trunk tergites [ZE2020-77]

(0) absent

(1) present

1. Simple head carapace, morphology [ZE2020-78]

(0) semi-circular

(1) heart-shaped

1. Bivalved carapace [ZE2020-79]

(0) absent

(1) present

1. Body length covered by bivalved carapace, length of anterior appendages excluded [ZE2020-80]

(0) > 70%

(1) 45–70%

(2) < 45%

1. Head carapace or shield, straight medial hinge [ZE2020-81]

(0) absent

(1) present

1. Valve shape of bivalved carapace [ZE2020-82]

(0) symmetrical respective to sagittal axis, ventral margins tight (‘*Isoxys* type’)

(1) asymmetrical respective to sagittal axis, anteroventral margin tight, posteroventral margin ample (‘*Canadaspis* type’)

(2) symmetrical respective to sagittal axis, ventral margins ample (‘*Branchiocaris* type’)

(3) symmetry variable, ventral margins ample (as in *Jugatacaris* and *Odaraia*)

1. Bivalved carapace, relative length of anterior spine compared to valve length [ZE2020-83]

(0) absent or tiny spines

(1) short spine

(2) long spine

1. Bivalved carapace, relative length of posterior spine compared to valve length [ZE2020-84]

(0) absent or tiny spines

(1) short spine

(2) long spine

1. Bivalved carapace, average length of valves in adults, anterior and posterior spines excluded [ZE2020-85]

(0) small, <2cm

(1) middle, 2-3.5 cm

(2) longer, > 4 cm

*Remark*: We here recode *Sunella* (reference herein), *O. oviformis*^10^, *F. valida*^11^, *Clypecaris*^13^ and *Waptia*^14^ as ‘0’. Code isoxyids as ‘1’, other hymenocarines as ‘2’.

1. Anterior sulcus [NEW]

(0) absent

(1) present

*Remark*: Typical for *Sunella*.

1. Bivalved carapace covering cephalothorax only [ZE2020-86]

(0) absent

(1) present

1. Bivalved carapace, posterior margin notched from dorsal view [ZE2020-87]

(0) absent

(1) present

1. Isoxyid-type bivalved carapace, curvature of anterior dorsal margin of valve [ZE2020-88]

(0) almost straight

(1) curved

1. Isoxyid-type bivalved carapace, relative lengths of anterior and posterior spines [ZE2020-89]

(0) posterior spine longer

(1) anterior spine longer

1. Soft-tissue bundle into the anterodorsal end of bivalved carapace [ZE2020-90]

(0) absent

(1) present

1. Fused head shield [ZE2020-91]

(0) absent

(1) present

1. Head shield articulated with reduced anterior trunk tergites [ZE2020-92]

(0) absent

(1) present

1. Transverse ridges or notches as segmental impression on head shield [ZE2020-93]

(0) absent

(1) present

1. Glabellar furrows or lobes [ZE2020-94]

(0) absent

(1) present

1. Anterior margin of head shield or carapace [ZE2020-95]

(0) convex

(1) almost straight

(2) subtriangular, rostral

(3) concave

1. Additional anterior marginal structures of head shield [ZE2020-96]

(0) absent

(1) pointed medially

(2) notched on single tergite

1. Genal angles of head shield [ZE2020-97]

(0) rounded genal angles

(1) acute genal angles

(2) genal spines

(3) spine-like extension

1. Medial notch on posterior margin of head shield [ZE2020-98]

(0) absent

(1) present

1. Doublure of head shield [ZE2020-99]

(0) absent or weak

(1) strong

1. Doublure of head carapace [ZE2020-100]

(0) absent or weak

(1) strong

1. Structure of protocerebrum [ZE2020-101]

(0) cycloneuralian brain or non-dorsally restricted ganglionic protocerebrum

(1) dorsal restriction of protocerebrum

1. Suboesophageal ganglion completely fused to thoracic plus abdominal ganglia [ZE2020-102]

(0) absent

(1) present

1. Stomodeum extends to rostral margin of protocerebrum [ZE2020-103]

(0) absent

(1) present

1. Number of segments in head region or covered by head shield or carapace [ZE2020-104]

(0) 1–2

(1) 3-4

(2) four and one-half

(3) 5

(4) ≥ 6

| **Trunk or thorax (non-appendicular structures)** |
| --- |

1. Metameres longest in middle trunk, shortening towards anterior and posterior [ZE2020-105]

(0) absent

(1) present

1. Number and form of annuli between legs per metamere on trunk [ZE2020-106]

(0) > 7, narrow

(1) < 6, wide

1. Dorsal/tergal boundary between head and thorax/trunk [ZE2020-107]

(0) absent

(1) present

1. Articulation between head and thorax/trunk non-functional [ZE2020-108]

(0) absent

(1) present

1. Occipital lobe [ZE2020-109]

(0) absent

(1) present

1. Trunk narrowed anteriorly relative to head shield [ZE2020-110]

(0) absent

(1) present

1. Dorsal/tergal boundary between thorax/trunk and pygidium/tail [ZE2020-111]

(0) absent

(1) present

1. Dorsal/tergal boundaries in thorax/trunk [ZE2020-112]

(0) absent

(1) present

1. Articulations of trunk tergites [ZE2020-113]

(0) tergites non-overlapping

(1) extensive overlap of tergites

(2) edge-to-edge pleural articulations

1. Trunk tergites weakly sclerotized [ZE2020-114]

(0) absent

(1) present

1. Articulating half rings on trunk segments [ZE2020-115]

(0) absent

(1) present

1. Thickened inter-segmental rims on both trunk segments and head shield [ZE2020-116]

(0) absent

(1) present

1. Straight cuticular ridge along with articulation between adjacent trunk segments [ZE2020-117]

(0) absent

(1) present

1. Tergal boundaries in trunk effaced [ZE2020-118]

(0) absent

(1) present

1. Posterior tergal articulations functional, anterior ones variably fused [ZE2020-119]

(0) absent

(1) present

1. Decoupling of multiple tergites and sternites/appendages in trunk [ZE2020-120]

(0) absent

(1) present

1. Anterior tergal boundaries in trunk or thorax reflexed anterolaterally [ZE2020-121]

(0) absent, boundaries traverse or reflexed posterolaterally

(1) present

1. Posterior tergites strongly curved compared to anterior tergites [ZE2020-122]

(0) absent

(1) present

1. Orientation of pleurae [ZE2020-123]

(0) horizontal

(1) around body

1. Constricted trunk pleural region [ZE2020-124]

(0) absent

(1) present

1. Size of pleurae [ZE2020-125]

(0) short, equal or inferior to body diameter

(1) long, exceeding body diameter

1. Trunk tergite, shape [ZE2020-126]

(0) rounded

(1) pleural tips

(2) extended, forming spines

1. Width change of trunk or thoracic tergites along the length [ZE2020-127]

(0) tapering or narrowing dramatically

(1) narrowing gradually, subequal widths

1. Tergo-pleural rings in trunk [ZE2020-128]

(0) absent

(1) present

1. Posterior trunk tergo-pleural rings strongly compacted, disc-like [ZE2020-129]

(0) absent

(1) present

1. Raised axial region [ZE2020-130]

(0) absent

(1) present

1. Axial furrows [ZE2020-131]

(0) absent

(1) present

1. Axial spine on non-terminal trunk tergite [ZE2020-132]

(0) absent

(1) present

1. Number of trunk divisions or tergites [ZE2020-133]

(0) 0–1

(1) 2–4

(2) 5–11

(3) 12–19

(4) 20–41

(5) ≥ 42

1. Number of prothoracic segments [ZE2020-134]

(0) 3

(1) 5 or 6

| **Posterior body** |
| --- |

1. Posterior trunk extension [ZE2020-135]

(0) absent

(1) present

1. Abdomen as posterior differentiated segments [ZE2020-136]

(0) absent

(1) present

1. Abdominal tergites narrowed in widths, distinguishing from thoracic tergites [ZE2020-137]

(0) absent

(1) present

1. Number of limbless posterior tergites [ZE2020-138]

(0) 0–2

(1) 3–5

(2) ≥ 6

1. Telson [ZE2020-139]

(0) absent

(1) present

1. Telson fringed with setae [ZE2020-140]

(0) absent

(1) present

1. Posterior tagmata with elongate lateral processes [ZE2020-141]

(0) absent

(1) present

1. Posterior tagmata composed of three paired tail flaps [ZE2020-142]

(0) absent

(1) present

1. Posteriormost lateral processes, morphology [ZE2020-143]

(0) absent

(1) present, isolated

(2) present, fused with middle projection

1. Posteriormost lateral processes fused [ZE2020-144]

(0) absent

(1) present

1. Posteriormost lateral processes recurved [ZE2020-145]

(0) absent

(1) present

1. Posteriormost lateral processes with lanceolate tips [ZE2020-146]

(0) absent

(1) present

1. Medial telson process [ZE2020-147]

(0) absent

(1) present

1. Telson shape [ZE2020-148]

(0) paddle-shaped or lanceolate

(1) rod-like

(2) flap-shaped, medial and paired lateral processes fused

(3) paired lateral processes unfused or incompletely fused

1. Posteriormost ovoid median plate attached to telson [ZE2020-149]

(0) absent

(1) present

1. Posteriormost tagma modified into a fluke [ZE2020-150]

(0) absent

(1) present

1. Multiple posterior segments fused, forming a pygidium [ZE2020-151]

(0) absent

(1) present

1. Pygidium, general shape [ZE2020-152]

(0) narrowed, bearing median spines

(1) ovoid to semi-circular

(2) widened, subrectangular, with lateral spines

1. Relative size of pygidium to cephalon [ZE2020-153]

(0) pygidium absent or micropygous

(1) subisopygous to isopygous

1. Median broad-based spine on pygidium [ZE2020-154]

(0) absent

(1) present

1. Lateral spines on pygidium [ZE2020-155]

(0) absent

(1) present

1. Caudal cerci [ZE2020-156]

(0) absent

(1) present

1. Tail flaps [ZE2020-157]

(0) absent

(1) present

1. Number of tail flap pairs [*ZE2020-158*]

(0) single

(1) two or three

(2) four or more

1. Furcae [ZE2020-159]

(0) absent

(1) present

| **Appendages (general)** |
| --- |

1. Paired appendages [ZE2020-160]

(0) absent

(1) present

1. Lobopodous appendages [ZE2020-161]

(0) absent

(1) present

1. Sclerotized appendages [ZE2020-162]

(0) absent

(1) present

1. Arthropodized appendages [ZE2020-163]

(0) absent

(1) present

1. Sclerotized head appendages [ZE2020-164]

(0) absent

(1) present

1. Arthropodized head appendages [ZE2020-165]

(0) absent

(1) present

1. Sclerotized trunk appendages [ZE2020-166]

(0) absent

(1) present

1. Arthropodized trunk appendages [ZE2020-167]

(0) absent

(1) present

| **Frontalmost appendages** |
| --- |

1. Frontalmost appendages, position on head [ZE2020-168]

(0) lateral

(1) dorsolateral

(2) ventral

1. Frontalmost appendages, orientation [ZE2020-169]

(0) non-specific or lateral

(1) downward

(2) upward

1. Frontalmost appendages, segmental identity [ZE2020-170]

(0) protocerebral

(1) deuterocerebral

1. Frontalmost appendages, arthropodization [ZE2020-171]

(0) absent

(1) present

1. Frontalmost appendages, annulation [ZE2020-172]

(0) absent

(1) present

1. Frontalmost appendages, composition of distal annuli or articulated podomeres, appendage terminal excluded [ZE2020-173]

(0) homonomous

(1) heteronomous

1. Frontalmost appendages, absolute number of distal articulated podomeres or annuli [ZE2020-174]

(0) smooth or ≥ 16

(1) 8–15

(2) 5–7

(3) 4

(4) 3

(5) ≤ 2

1. Frontalmost appendages, proximal shaft region differentiated from distal articulated podomeres [ZE2020-175]

(0) absent

(1) present

1. Relative number of podomeres or annuli in frontalmost appendages compared to that in trunk endopods or legs [ZE2020-176]

(0) close

(1) significantly more

(2) significantly less

(3) reduced

1. Frontalmost appendages specialised, with a unique morphology compared to all other appendages [ZE2020-177]

(0) absent

(1) present

1. Frontalmost appendages, antenniform [ZE2020-178]

(0) absent

(1) present

Remark: According to Fu et al.^15^ and Stein et al.^16^, we here recode *I. auritus* and *I. volucris* as present, and other isoxyids as absent

1. Frontalmost appendages, endites well-developed, raptorial [ZE2020-179]

(0) absent

(1) present

1. Frontalmost appendages, terminal structures [ZE2020-180]

(0) similar to other podomeres

(1) claw

(2) cuticular spines

1. Frontalmost appendages, terminal claw bearing multiple cusps [ZE2020-181]

(0) absent

(1) present

1. Frontalmost appendages (unarthropodized), located at the protruded ‘neck’ [ZE2020-182]

(0) absent

(1) present

1. Frontalmost appendages (unarthropodized), antenniform [ZE2020-183]

(0) absent

(1) present

1. Frontalmost appendages (unarthropodized), tentacle-like [ZE2020-184]

(0) absent

(1) present

1. Frontalmost appendages (unarthropodized), unspecialised lobopod [ZE2020-185]

(0) absent

(1) present

1. Frontalmost appendages (unarthropodized), specialised raptorial lobopod [ZE2020-186]

(0) absent

(1) present

1. Frontalmost appendages (unarthropodized), non-terminal spinous outgrowths [ZE2020-187]

(0) absent or invisible

(1) present

1. Frontalmost appendages (arthropodized), number of podomeres bearing well-expanded endites, terminal podomere excluded [ZE2020-188]

(0) 0 or 1

(1) 2 or 3

(2) 4–7

(3) ≥ 8

1. Frontalmost appendage, separated into a proximal shaft (base) and a distal claw [NEW]

(0) absent

(1) present

Remark: Present in Radiodonta, *Kylinxia*, *Sunella* and Megacheira. Flowing to O’Flynn et al.^12, 17^, we recode *Fengzhengia* as ‘?’, since there no details of proximal region of frontal appendages.

1. Frontalmost appendage, the angular range of the dorsal surface between the base and claw [NEW]

(0) <90°

(1) >90°

Remark: An obtuse angle showing in Radiodonta and *Sunella*, differented from that of Megacheira.

1. Frontalmost appendage: Base: number of podomere(s) [WU2024_69]

(0) one

(1) two

(2) three

Remark: In almost hurdiids, the base is composed of one podomere, and thus coded as (0).

1. Frontalmost appendage: podomere number of the long distal claw [NEW]

(0) <9

(1) 9 to 11

(2)12 to 15

1. Frontalmost appendages (arthropodized), shaft endite [ZE2020-189]

(0) absent

(1) present

1. Frontalmost appendages (arthropodized), differentiated main spines or main protrusions of endites [ZE2020-190]

(0) absent

(1) present

1. Frontalmost appendages (arthropodized), pair of endites on claw podomere [NEW]

(0) absent

(1) present

Remark: A feature present in Radiodonta, and *Sunella*.

1. Frontalmost appendages (arthropodized), relative length of endite to podomere height [ZE2020-191]

(0) absent or shorter

(1) comparable

(2) much longer

1. Frontalmost appendages (arthropodized), relative width of base of endite to podomere length [ZE2020-192]

(0) endite absent or narrowed

(1) comparable, along the entire podomere

1. Frontalmost appendages (arthropodized), morphology of enditic spines [ZE2020-193]

(0) absent

(1) spiky

(2) elongated, blade-like

(3) elongated, finger-like

(4) needle-like

1. Frontalmost appendages (arthropodized), enditic spines originated from distal portion of podomere, tapering distally [ZE2020-194]

(0) absent

(1) present

1. Frontalmost appendages (arthropodized), endites broadened laterally, overlapping with adjacent ones [ZE2020-195]

(0) absent

(1) present

1. Frontalmost appendages (arthropodized), endites, change of lengths along the appendage [ZE2020-196]

(0) decreasing gradually towards tip

(1) alternating (short-long-short etc)

1. Frontalmost appendages (arthropodized), endite pincer-like [ZE2020-197]

(0) absent

(1) present

1. Frontalmost appendages (arthropodized), endites bearing well-developed auxiliary spines [ZE2020-198]

(0) absent

(1) present

1. Frontalmost appendages (arthropodized), endites, auxiliary spines distribution [ZE2020-199]

(0) both anterior and posterior

(1) anterior only

1. Frontalmost appendages (arthropodized), chelate or sub-chelate endites on distal podomeres [ZE2020-200]

(0) absent

(1) present

1. Frontalmost appendages (arthropodized), length of basal podomere(s) [ZE2020-201]

(0) short

(1) elongated

1. Frontalmost appendages (arthropodized), oblique arthrodial membrane in shaft region [ZE2020-202]

(0) absent

(1) present

1. Frontalmost appendages (arthropodized), proximal podomeres differentiated and peduncle-like [ZE2020-203]

(0) absent

(1) present

1. Frontalmost appendages (arthropodized), peduncle podomere morphology [ZE2020-204]

(0) short

(1) elongated

1. Frontalmost appendages (arthropodized) geniculate at the middle pivot, forming an elbowed articulation [ZE2020-205]

(0) absent

(1) present

1. Frontalmost appendages (arthropodized), elongate terminal podomere [ZE2020-206]

(0) absent

(1) present

1. Frontalmost appendages (arthropodized), flagella [ZE2020-207]

(0) absent

(1) present

1. Frontalmost appendages (arthropodized), length of flagella [ZE2020-208]

(0) midlength of trunk

(1) end of body

1. Frontalmost appendages (arthropodized), dorsal spines [ZE2020-209]

(0) absent or highly reduced

(1) present

1. Frontalmost appendages (arthropodized), length of multi-podomerous antenniform types compared with length of bivalved head carapace [ZE2020-210]

(0) short

(1) long

1. Frontalmost appendages (arthropodized), raptorial device made of three to four podomeres with elongate endites [ZE2020-211]

(0) absent

(1) present

1. Frontalmost appendages (arthropodized), chelate endites only on terminal podomeres, cheliceralike or chelicerae [ZE2020-212]

(0) absent

(1) present

| **Post-oral appendages** |
| --- |

1. Anteriormost appendages differentiated from posterior ones [ZE2020-213]

(0) absent

(1) present

1. Differentiated multiple pairs of anterior homonomous unarthropodized appendages [ZE2020-214]

(0) absent

(1) present

1. Differentiated multiple pairs of anterior homonomous unarthropodized appendages, number of pairs [ZE2020-215]

(0) two to three

(1) five to six

1. Differentiated multiple pairs of anterior homonomous unarthropodized appendages, bearing spine-like long claws [ZE2020-216]

(0) absent

(1) present

1. Multiple post-oral homonomous appendages (legs, flaps) accommodated to head region, differentiated from the frontalmost and other post-oral appendages [ZE2020-217]

(0) absent

(1) present

1. Anterior homonomous appendages in head smaller, differentiated in size from posterior ones [ZE2020-218]

(0) absent

(1) present

1. Anterior reduction of segments and/or appendages [ZE2020-219]

(0) absent

(1) present

1. Second antennae on third head segment [ZE2020-220]

(0) absent

(1) present

1. Specialised post-antennal appendages (SPAs) [ZE2020-221]

(0) absent

(1) present

1. Morphology of appendages on third head segment [ZE2020-222]

(0) homonomous to posterior appendages

(1) heteronomous to posterior appendages, or reduced, forming intercalary segment

1. Mandibles or paired mandible-like appendages [ZE2020-223]

(0) absent

(1) present

1. Mandible morphology [ZE2020-224]

(0) basipodite with elaboration of proximal endite

(1) coxal endite embedded between the labrum and hypopharynx to form a chewing chamber

1. Mandibular palp [ZE2020-225]

(0) absent

(1) present

1. Specialised raptorial post-tritocerebral appendages [ZE2020-226]

(0) absent

(1) present

1. Number of unique morphological types of anteriormost appendage pairs [ZE2020-227]

(0) 0

(1) 1

(2) 2

(3) ≥ 3

1. Differentiation of trunk appendages in sets, each consisting of multiple appendages in similar morphology [ZE2020-228]

(0) absent

(1) present

1. External metameric boundaries on unarthropodized trunk appendages [ZE2020-229]

(0) absent

(1) present

1. Well-developed papillae or cuticular outgrowth on unsclerotized appendages [ZE2020-230]

(0) absent

(1) present

1. Post-oral appendages, length [ZE2020-231]

(0) subequal along length of body

(1) anterior appendages about twice as long as posterior appendages

1. Post-oral telescopic legs or lobopods [ZE2020-232]

(0) absent

(1) present

1. Post-oral telescopic legs or lobopods, multiple rows of cuticular spines in feather-like arrangement [ZE2020-233]

(0) absent

(1) present

1. Post-oral telescopic legs or lobopods bearing long setiform spines [ZE2020-234]

(0) absent

(1) present

1. Post-oral telescopic legs or lobopods, relative widths [ZE2020-235]

(0) very narrowed

(1) narrower than trunk diameter

(2) comparable to trunk diameter

1. Post-oral telescopic legs or lobopods, relative length [ZE2020-236]

(0) shorter or close to trunk diameter

(1) at least 1.5 times longer than trunk diameter

(2) state 0 or state 1

1. Number of leg pairs on trunk terminal [ZE2020-237]

(0) one

(1) two

1. Papillae or spines on legs [ZE2020-238]

(0) absent or tiny

(1) prominent

1. Number of claw elements on leg or frontalmost appendage [ZE2020-239]

(0) none

(1) single

(2) double

(3) multiple

1. Stacked structure of claws or sclerites [ZE2020-240]

(0) absent

(1) present

1. Large distal claw elements with a wide base on appendages [ZE2020-241]

(0) absent

(1) present

1. Number of claws on posteriormost lobopods [ZE2020-242]

(0) multiple

(1) single

1. Posteriormost claws or legs orientated to the anterior [ZE2020-243]

(0) absent

(1) present

1. Post-oral appendages, protopodite or gnathobase-like structures [ZE2020-244]

(0) absent

(1) present

1. Protopodite or gnathobase, fusion of multiple podomeres [ZE2020-245]

(0) superficial repetitive structures invisible

(1) repetitive homonomous enditic structures visible

(2) multiple segmented podomeres and boundaries visible

1. Protopodite, proximal endite [ZE2020-246]

(0) absent

(1) present

1. Protopodite, coxa [ZE2020-247]

(0) absent

(1) present

1. Biramous appendages with endopodites and exopodites [*ZE2020-248*]

(0) absent

(1) present

1. Post-oral appendages, jointed legs or endopodites [ZE2020-249]

(0) absent

(1) present

1. Post-oral appendages, endopodite or leg, proximal-distal differentiation of podomeres or annuli towards tip [ZE2020-250]

(0) more or less homonomous, tapering continuously

(1) differentiated, discontinuous change in length-width ratio of podomeres

1. Post-oral appendages, endopodite, endites [ZE2020-251]

(0) absent or well-reduced

(1) distal spines only

(2) serrated rows of spines

(3) well-extended endite with a cluster of multiple spines

1. Post-oral appendages, endopodite, number of podomeres with distal claw included [ZE2020-252]

(0) ≥ 12

(1) 8–11

(2) 7

(3) ≤ 6

1. Post-oral appendages, endopodite, chelate terminal podomere [ZE2020-253]

(0) absent

(1) present

1. Post-oral appendages, endopodite and protopodite as a whole, maximum number of podomeres [ZE2020-254]

(0) ≥ 13

(1) 9–12

(2) 8

(3) ≤ 7

1. Protopodite and endopodite podomeres close in lengths [ZE2020-255]

(0) absent

(1) present

1. Flaps or exopodites [ZE2020-256]

(0) absent

(1) present

1. Flaps or exopodites, attachment [ZE2020-257]

(0) body flap

(1) protopodite only

1. Exopodite morphology, flaps [ZE2020-258]

(0) absent

(1) present

1. Exopodite morphology, lobe shape [ZE2020-259]

(0) lobe absent

(1) undivided lobe

(2) shorter proximal lobe and longer distal lobe

(3) proximal lobe no shorter than distal lobe

1. Exopodite morphology, segmented podomeres [ZE2020-260]

(0) absent

(1) present

1. Exopodite morphology, numerous annuli [ZE2020-261]

(0) absent

(1) present

1. Multiple exopodite lobes or book gills [ZE2020-262]

(0) absent

(1) present

1. Exopodite differentiated into proximal lobe bearing imbricated lamellar setae and distal lobe fringed by non-lamellar setae [ZE2020-263]

(0) absent

(1) present

1. Proximal lobe of exopodite [ZE2020-264]

(0) flattened lobe

(1) narrowed lobe or shaft

1. Distal lobe of exopodite [ZE2020-265]

(0) large

(1) small to moderate size

1. Flaps or exopodites, anterior and posterior parts differentiation [*ZE2020-266*]

(0) absent

(1) present

1. Flaps or exopodites, internal strengthening [*ZE2020-267*]

(0) absent

(1) present

1. Exopodites, septa or narrowed proximal part [ZE2020-268]

(0) absent

(1) present

1. Differentiation of setae along exopodite [ZE2020-269]

(0) absent

(1) present

1. Trunk exopodite, setae [ZE2020-270]

(0) absent or short fine setae

(1) present

1. Trunk exopodite, slightly separated oblanceolate setae [ZE2020-271]

(0) absent

(1) present

1. Trunk exopodite, lamellar setae [ZE2020-272]

(0) absent

(1) present

1. Trunk exopodite, widely spaced filamentous setae [ZE2020-273]

(0) absent

(1) present

1. Non-lamellar marginal setae on exopodite [ZE2020-274]

(0) absent

(1) present

1. Setal structures [ZE2020-275]

(0) absent

(1) present

1. Setal structures, distribution [ZE2020-276]

(0) confined laterally, associated with lateral flaps or exopodites

(1) present dorsally

1. Setal structures, fusion [ZE2020-277]

(0) unfused

(1) fused across the back

1. Imbrication of setal structures [ZE2020-278]

(0) absent

(1) present

1. Epipodite [ZE2020-279]

(0) absent

(1) present

1. A series of long internal soft structures deep into trunk appendages [ZE2020-280]

(0) absent

(1) present

1. Lengths of appendages subequal along middle trunk, shortening towards anterior and posterior [ZE2020-281]

(0) absent

(1) present

1. Appendages behind the third pair of appendages, morphology [ZE2020-282]

(0) homonomous, undifferentiated

(1) heteronomous, differentiated into various types

1. Number of appendage pairs [ZE2020-283]

(0) ≤ 8

(1) 9–10

(2) 11–14

(3) 15 or 16

(4) ≥ 17

**SUPPLEMENTARY REFERENCES**

1. Zeng, H., Zhao, F., Niu, K, Zhu, M. & Huang, D. An early Cambrian euarthropod with radiodont-like raptorial appendages. *Nature* **588**, 101–105 (2020).
2. Wu, Y. et al. A new radiodont from the lower Cambrian (Series 2 Stage 3) Chengjiang Lagerstätte, South China informs the evolution of feeding structures in radiodonts, *J. Syst. Palaeontol.* **22**(1), 2364887 (2024).
3. Zhang, C. et al. Three-dimensional morphology of the biramous appendages in *Isoxys* from the early Cambrian of South China, and its implications for early euarthropod evolution. *Proc. R. Soc. B* **290**: 20230335 (2023).
4. Daley, A.C. & Edgecombe, G.D. Morphology of Anomalocaris canadensis from the Burgess Shale. *J. Paleontol.* **88**, 68–91 (2014).
5. Cong, P., Daley, A.C., Edgecombe, G.D., Hou, X. & Chen, A. Morphology of the radiodontan *Lyrarapax* from the early Cambrian Chengjiang biota. *J. Paleontol.* **90**(4), 663–671 (2016).
6. Cong, P. et al. Brain structure resolves the segmental affinity of anomalocaridid appendages. *Nature* **513**, 538–542 (2014).
7. Kühl, G., Brigss, D.E.G. & Rust, J. A Great-Appendage Arthropod with a Radial Mouth from the Lower Devonian Hunsrück Slate, Germany.*Science* **323**,771-773 (2009).
8. Daley, A.C., Budd, G.E. & Caron, J.-B. Morphology and systematics of the anomalocaridid arthropod Hurdia from the Middle Cambrian of British Columbia and Utah. *J. Syst. Palaeontol.* **11**: 743–787 (2013).
9. Aria, C. & Caron, J.-B. Cephalic and limb anatomy of a new isoxyid from the Burgess Shale and the role of ‘stem bivalved arthropods’ in the disparity of the frontal most appendage. *PLoS ONE* **10**, e0124979 (2015).
10. Hou, X. New rare bivalved arthropods from the Lower Cambrian Chengjiang fauna, Yunnan, China. *J. Paleontol.* **73**, 102–116 (1999).
11. Yang, J., Ortega-Hernández, J., Lan, T., Hou, J. & Hou, X. A predatory bivalved euarthropod from the Cambrian (Stage 3) Xiaoshiba Lagerstätte, South China. *Sci. Rep.* **6**, 27709 (2016).
12. O’Flynn, R.J. et al. The early Cambrian *Kylinxia zhangi* and evolution of the arthropod head. *Current Biology* **33**, 1–8 (2023).
13. O’Flynn, R.J. et al. A new euarthropod with ‘great appendage’-like frontal head limbs from the Chengjiang Lagerstätte, Southwest China. *Palaeontol. Electronica* **23**(2): a36 (2020).
14. Vannier, J., Aria, C., Taylor, R.S. & Caron, J.B. *Waptia fieldensis* Walcott, a mandibulate arthropod from the middle Cambrian Burgess Shale. *R. Soc. Open Sci.* **5**(172206) (2018).
15. Fu, D., Zhang, X., Budd, G.E., Liu, W. & Pan, X. Ontogeny and dimorphism of *Isoxys auritus* (Arthropoda) from the early Cambrian Chengjiang biota, South China. *Gondwana Res.* **25**, 975–982 (2014).
16. Stein, M., Peel, J.S., Siveter, D.J. & Williams, M. *Isoxys* (Arthropoda) with preserved soft anatomy from the Sirius Passet Lagersätte, Lower Cambrian of North Greeland. *Lethaia* **43**, 258-265 (2010).
17. O’Flynn, R.J. et al. The early Cambrian *Bushizheia* yangi and head segmentation in upper stem-group euarthropods. *Pap. Palaeontol.* e1556 (2024).
